# Resolving conflicts during sentence processing in L1 and L2: eye-tracking evidence from gender stereotypes and zero plurals

**DOI:** 10.3389/fpsyg.2026.1913837

**Published:** 2026-10-27

**Authors:** Denisa Bordag, Andreas Opitz, Hans-Georg Berulava

**Affiliations:** 1 Herder-Institut, Universität Leipzig, Leipzig, Germany; 2 University of Haifa, Haifa, Israel

**Keywords:** eye tracking, reading, L1-L2 differences, German, gender stereotypes, grammatical gender, zero plurals, morphosyntactic agreement

## Abstract

This eye-tracking study examined how L1 and L2 readers of German resolve two sources of processing difficulty: a conceptual–lexical conflict between gender stereotypes and masculine/feminine role nouns, and a formal–structural conflict induced by zero-marked plurals that are morphologically identical to singular forms. Sixty-nine L1 German speakers and 70 advanced L2 learners read sentences manipulating (i) role-noun gender bias (balanced/male-biased) × grammatical gender form (masculine/feminine) and (ii) plural marking (overt/zero) × number (singular/plural). In early measures (gaze duration), feminine forms of male-biased role nouns increased first-pass reading times in both groups, with no corresponding increase in regressions, suggesting local resolution during lexical access. By contrast, zero-marked plurals affected later processing: in plural contexts, ambiguous plurals triggered more regressions and longer rereading, especially in L2 readers, indicating protracted agreement integration and reanalysis when an overt number cue on the noun is absent. These findings dissociate conceptual–lexical and morphosyntactic conflict in L1/L2 sentence processing.

## 1 Introduction

Language comprehension requires readers to rapidly integrate multiple sources of information, including conceptual knowledge associated with lexical items and formal grammatical cues that structure the sentence. These sources of information can occasionally give rise to processing difficulty when different cues support competing interpretations. Examining how such conflicts are resolved provides important insights into the mechanisms that support language comprehension.

In the current study, we explore how readers resolve conceptual–lexical and formal–structural conflicts when processing grammatical features during sentence reading, and whether the mechanisms involved in resolving these two types of conflict differ between L1 and L2 processing. To address this research question, we employed gender and number of role nouns (i.e., personal nouns referring to social or professional roles) in German, which allows us to operationalize both types of conflict.

Conceptual–lexical conflict was instantiated in gender-marked noun phrases consisting of a definite article and a role noun in either masculine or feminine singular form in subject position of a sentence. We manipulated the lexical gender bias of the role nouns. In the balanced condition, the masculine and the feminine forms (e.g., *der Lehrer* ‘the.M teacher.M’ vs. *die Lehrerin* ‘the.F teacher.F’) did not conflict with conceptual–lexical knowledge about the stereotypical sex of their referents. In the biased condition, however, the role nouns were lexically associated with a primarily male interpretation. In these cases, the morphologically expressed grammatical gender either aligned with (e.g., *der Pilot* ‘the.M pilot.M’) or conflicted with (e.g., *die Pilotin* ‘the.F pilot.F’) the stereotypical gender associated with the role, thereby inducing conceptual–lexical conflict during processing.

Formal–structural conflict was operationalized through plural number marking in the subject noun phrase and the corresponding verb. We manipulated the morphological realization of plural marking on the role nouns. In our study, we employed an overt plural condition in which the role nouns expressed plural through an overt plural suffix (e.g., *Fotograf* – *Fotografen* ‘photographer.SG – photographers.PL’), whereas in the zero-plural condition the plural forms were formally identical to the singular form (e.g., *Lehrer* – *Lehrer* ‘teacher – teachers’). In the latter case, number interpretation relies more strongly on syntactic agreement cues than on overt morphological marking, because the noun form itself is morphologically ambiguous between singular and plural. We refer to this situation as a formal–structural conflict. It is not a conflict between incompatible agreement cues: all agreement cues in the sentence consistently indicate plural. Rather, the conflict is grounded in the ambiguous morphological form of the noun, which is identical to the singular and thus provides no explicit plural marking, whereas the sentence-level agreement cues require a plural interpretation. In this sense, both manipulations involve a conflict between two sources of information about the same feature: in the gender manipulation, between the morphologically marked gender and the stereotypical gender associated with the role noun; in the number manipulation, between a noun form identical to the singular and the plural number indicated by the agreement cues.

The L1s of the L2 participants were Slavic languages (Czech, Ukrainian and Russian) that exhibit conceptual–lexical and formal–structural patterns closely comparable to those in German. They mark grammatical gender on role nouns via derivational suffixes and allow for both overt and zero plural marking in certain noun classes (see also below). Furthermore, grammatical gender and number are also expressed morphologically via agreement, for instance on dependent determiners and adjectives. This typological similarity enables us to examine L2 processing in a context in which the relevant grammatical and conceptual mappings are largely shared across the two languages. Consequently, potential L1–L2 differences cannot be attributed to structural differences between the languages, but rather to more general differences in L1 and L2 processing mechanisms (see Sato et al., 2013).

Research across languages such as English, French, and German suggests that readers infer the biological gender of nouns denoting human referents even when it is not strictly necessary for local coherence (Gygax et al., 2021). Stereotypical knowledge associated with role nouns is known to be activated rapidly and automatically during comprehension (Banaji and Hardin, 1996; Carreiras et al., 1996; Garnham et al., 2002; Molinaro et al., 2016), often influencing the interpretation of gender even before later contextual information becomes available. Frameworks such as the resonance model (Myers and O’Brien, 1998) or its extension, the RI-Val (Resonance-Integration-Validation; Cook and O’Brien, 2014) have been applied to explain how role nouns send signals to long-term memory that trigger associated stereotypical knowledge to resonate, i.e., to become activated in a passive, automatic manner in response to those signals (e.g., Lassonde, 2015). While this inference is driven primarily by social stereotypes in natural gender languages like English (and can be constrained by competitive contexts or via discourse coherence), it is further modulated by formal grammatical markers in languages like German and French (Sato et al., 2013). In such grammatical gender languages, gender is a morphosyntactic property encoded in the lexical representation of nouns and systematically expressed through agreement on, e.g., determiners, adjectives, and pronouns (Corbett, 1991; Barber and Carreiras, 2005; Kramer, 2016).

Research on gender bias has been primarily addressed in studies investigating how masculine generics are interpreted during language comprehension. Work on pronoun resolution and referent interpretation has demonstrated that stereotypical gender information is rapidly integrated into the mental representation of discourse during comprehension (Carreiras et al., 1996; Duffy and Keir, 2004; Gygax et al., 2021) and shapes readers’ expectations about the referent. Across languages (German, French, English, Norwegian, Spanish, etc.) studies consistently show that female continuations are processed more slowly or judged as less plausible following masculine generics, suggesting that grammatically masculine forms tend to activate male-specific representations rather than gender-neutral ones (e.g., Anaya-Ramírez et al., 2022; Braun et al., 2005; Gabriel et al., 2017; Gygax et al., 2009; Irmen and Roßberg, 2004). Research on bilingual comprehension further indicates that gender representations can shift depending on the language being processed. For example, balanced bilingual speakers rely more strongly on stereotypical information in languages without grammatical gender marking, whereas grammatical gender cues play a larger role in languages where gender is morphologically encoded (Sato et al., 2013).

Importantly, while gender bias in role nouns has been extensively studied in L1 comprehension and in a few bilingual studies, all previous L2 research has focused on the processing of grammatical gender without addressing how conceptual–lexical gender information interacts with lexical access in L2. The only recent exception is a study by Jankowiak et al. (2025): Polish–English unbalanced bilinguals completed a task in which they judged the meaningfulness of sentences presented in their L1 and L2 while their brain activity was recorded using EEG. The sentences were either stereotypically congruent or incongruent, and either semantically correct or incorrect. The results provided evidence for a foreign language effect (FLE) in L2 processing. In addition to reducing emotional reactivity (e.g., Hayakawa et al., 2017), the FLE may also attenuate responses to gender-related stereotypes in stereotype-incongruent contexts, albeit this was observed only for a subgroup of male participants in their study. While female participants processed stereotype-congruent and stereotype-violating sentences similarly in both languages, male participants showed clear stereotype effects in their L1 but reduced sensitivity in the L2. However, the results may also be partially connected to the participants’ L1, namely Polish, in combination with their L2 English used in the experimental items. The two languages differ greatly in the way they encode gender, as Polish is a grammatical gender language, whereas English belongs to the category of natural gender languages. Therefore, the observed effect might not be an FLE per se but possibly an outcome of typological differences in the L1–L2 pairing and the fact that the more pronounced gender encoding in Polish makes gender bias more accessible in the L1.

The present study addresses this empirical gap by examining stereotype activation among L2 speakers whose Slavic L1s share relevant aspects of grammatical gender encoding with German. The L2 group consisted primarily of Czech speakers, alongside Russian and Ukrainian speakers; all three L1s, like German, have grammatical gender systems that distinguish masculine, feminine, and neuter categories. This allows us to determine whether mechanisms of stereotype activation and conflict resolution observed in L1 also operate in a non-native language context when the L1 and L2 are broadly comparable with respect to the gender categories. This focus not only extends the existing work on gendered role nouns to L2 comprehension but also strengthens the theoretical claims about the universality and flexibility of stereotype-driven processing across languages. Eye tracking is employed for the first time to study online language processing in this context.

In contrast to previous L1 studies, the present study further differs in two important respects. First, rather than focusing on masculine generics, we examine the processing of explicit feminine derivatives of role nouns that differ in their lexical gender bias, comparing feminine forms derived from male-biased nouns (e.g., *Technikerin* ‘technician.F’) with those derived from gender-balanced nouns (e.g., *Kollegin* ‘colleague.F’). Second, we do not examine the effects of gender stereotypes on subsequent referential processing, for instance, when readers encounter gender-marked pronouns that either match or violate stereotypical expectations (e.g., Kreiner et al., 2008). Instead, we investigate how gender bias affects the processing of the noun (phrase) itself during initial lexical access and whether conflict resolution at the noun phrase stage influences later sentence processing. Investigating gender effects directly at the noun is crucial because stereotypical gender information and grammatical gender cues are initially established at this point in the sentence, before later agreement relations are computed (Popov et al., 2020).

In contrast to gender, which is an inherent and fixed property of the noun and retrieved during lexical access (Bock and Levelt, 1994; Corbett, 1991; Harris, 1991), number is a variable conceptual, or notional, feature signaling the quantity of the referent (Bock and Miller, 1991; Barber and Carreiras, 2005; Chondrogianni, 2024). Number typically adopts at least two different forms, singular and plural. Rather than being fixed at the lemma stage (Faussart et al., 1999), number is processed during postlexical integration and viewed as the syntactic head of an independent functional projection (Ritter, 1988, 1993). Consequently, number processing in the sentential context has primarily been investigated in research on morphosyntactic agreement. Most previous work has examined agreement violations or attraction effects at the verb. Agreement violations reliably lead to increased reading times, regressions, and electrophysiological responses associated with syntactic reanalysis (e.g., Fuchs et al., 2025; Hagoort et al., 1993; Lim and Christianson, 2015; Osterhout and Mobley, 1995; Zawiszewski et al., 2016). Studies using the number attraction paradigm further show that readers sometimes accept or process incorrect agreement when an interfering plural distractor is present (e.g., Chromý et al., 2026; Lago and Felser, 2018; Laurinavichyute and von der Malsburg, 2022; Wagers et al., 2009). Together, these findings indicate that number features are rapidly integrated into the evolving syntactic representation and play an important role in guiding the establishment of grammatical dependencies during sentence processing.

Further research comparing processing of number and gender has reported selective reactivation of number-matching, but not gender-matching antecedents (De Vincenzi, 1999; De Vincenzi and Di Domenico, 1999), as well as stronger gender than number incongruency effects (Faussart et al., 1999). Similarly, ERP studies have shown greater processing demands for gender than number, as reflected in larger late P600 effects (Barber and Carreiras, 2003, 2005). However, the relative processing demands may be influenced by L1–L2 transfer and L2 proficiency (Alemán Bañón et al., 2018; Dowens et al., 2011; Gabriele et al., 2013).

Contemporary models of sentence comprehension assume that agreement computation is guided by morphosyntactic feature information that becomes incrementally available during processing (e.g., Lewis and Vasishth, 2005; McElree et al., 2003; Wagers et al., 2009). Within this perspective, the reliability and salience of morphological cues may influence processing efficiency. Overt morphological marking provides an explicit cue to number interpretation, whereas forms without overt marking can introduce temporary ambiguity and require readers to rely more strongly on other agreement cues within the sentence. However, most previous research has examined these processes in contexts involving clear agreement violations or competing agreement controllers. Consequently, little is known about how differences in the morphological realization of number on the noun itself influence sentence processing when agreement is grammatically correct, an issue that the present study addresses.

Differences in the availability of morphological cues may be particularly relevant in L2 sentence processing. Several influential accounts propose that L2 comprehenders rely more strongly on overt surface cues and less on predictive structural processing than L1 readers (Clahsen and Felser, 2006, 2018; Bates and MacWhinney, 1989). If this is the case, morphologically ambiguous forms could pose greater challenges in L2 because number information must be inferred from the surrounding syntactic context rather than directly from the lexical form. Empirical work on L2 agreement processing further suggests that sensitivity to gender and number agreement develops gradually and may depend on factors such as proficiency and working memory capacity (e.g., Sagarra and Herschensohn, 2010; Lemmerth and Hopp, 2019).

The crucial aspect of the two main manipulations in our study is that they target different sources of processing difficulty. Conceptual–lexical conflict arises from a mismatch between morphological gender marking and the stereotypical gender associated with the role noun. Because gender bias is part of the lexical–conceptual representation of the role noun, such conflicts are expected to arise during lexical access, when readers retrieve both the morphologically encoded gender and the stereotypical gender information associated with the lexical item. In contrast, formal–structural conflict results from ambiguity in number marking and primarily concerns the availability of morphosyntactic information required to establish agreement within the sentence. In these cases, readers must rely more strongly on agreement cues elsewhere in the sentence to determine the correct number interpretation, which may place greater demands on morphosyntactic integration processes.

By comparing these two types of conflict within a single paradigm, the present study allows us to examine whether conceptual–lexical information accessed at the level of the lexical entry (gender) and formal–structural information involved in agreement computation (number) give rise to similar or distinct processing profiles during sentence comprehension in L1 and L2. We hypothesize that conflicts involving gender bias, which arise from conceptual–lexical information associated with the role noun and reflect non-linguistic, socially grounded stereotype knowledge, will be resolved in a similar manner in L1 and L2 readers, despite limited recent evidence from offline measures suggesting bilinguals may differ in explicit stereotype activation (Castro et al., 2026). This hypothesis rests on the assumption that stereotypical role knowledge is part of general world knowledge rather than of the language system itself and that its activation is passive and automatic (Myers and O’Brien, 1998; Cook and O’Brien, 2014), hence largely independent of the constraints that distinguish L1 from L2 processing. Language-dependent shifts in stereotype reliance have been observed where the languages differ in gender encoding (Sato et al., 2013), which is not the case here, and influential accounts of L1–L2 differences concern structural computation rather than lexical–conceptual retrieval (Clahsen and Felser, 2006). The formal–structural conflict resulting from ambiguity in number marking, by contrast, is tied more directly to the computation of morphosyntactic agreement relations within the sentence. Because such processes depend on the efficient use of grammatical cues during sentence integration, they may be more susceptible to differences between native and non-native processing mechanisms.

## 2 Methods

The experiments were part of a larger eye-tracking study with multiple subexperiments. Here we report only the Methods relevant to the critical materials used to test gender bias and zero-marked plurals.

### 2.1 Participants

A total of 144 participants took part, but 5 (2 L2, 3 L1) were excluded prior to analysis due to insufficient recording quality. The final sample comprised 69 L1 German speakers (*M* age = 26.0 years, *SD* = 4.73, range = 18–40) and 70 L2 German learners with a Slavic L1 (54 Czech, 8 Russian, 8 Ukrainian; *M* age = 23.9 years, *SD* = 4.2, range = 19–40) at B2/C1 level. The L2 participants had grown up in the Czech Republic, Russia, and Ukraine, respectively. All participants had normal or corrected-to-normal vision, reported no reading/cognitive impairments, and had no relevant neurological conditions. Participants provided consent and received monetary compensation. After eye tracking, participants completed a modified LexTALE vocabulary test and a visual digit span test of non-verbal short-term memory.^1^ Participants additionally completed a language background questionnaire. In addition to the LexTALE scores, the German proficiency of L2 participants was also self-assessed by participants according to the CEFR (Common European Framework of Reference for Languages). A summary of all participants’ scores is given in Table 1.

**Table 1:** Participant characteristics by group.

Characteristic	L1 group	L2 group
*N*	69	70
Age in years, M (SD)	25.96 (4.73)	23.93 (4.20)
Age range (years)	18–40	19–40
Sex (female / male / diverse)	59/7/3	62/8/0
Native language	German (69)	Czech (54), Russian (8), Ukrainian (8)
Proficiency in German (CEFR)	native	C1: 50, B2: 20
LexTALE (%), *M* (*SD*)	95.74 (3.28)ᵃ	81.85 (7.40)ᵇ
LexTALE range (%)	85.00–100.00	66.25–96.25
Digit span, *M* (*SD*)	6.12 (1.08)	5.93 (0.97)ᶜ
Digit span range	4–8	4–8

CEFR = Common European Framework of Reference for Languages. ᵃ *n* = 47. ᵇ *n* = 67. ᶜ*n* = 69. LexTALE scores were not available for all participants.

All participants (L1 and L2) reported intermediate to advanced proficiency of English as a second language. Further knowledge of non-native languages was heterogeneous within both L1 and L2 participants with respect to language and proficiency.^2^ None of the participants (L1 and L2) were balanced bilinguals.

### 2.2 Materials

We constructed 32 experimental items, each built around a German role noun selected on the basis of several pretests (see Supplementary Material for details). The nouns were chosen so that two properties were fully crossed: gender bias (16 gender-balanced vs. 16 male-biased nouns) and plural type (16 zero-plural vs. 16 overt-plural nouns), resulting in eight nouns per combination. The same 32 items therefore entered both the gender and the number manipulation, grouped according to the respective property.

Gender-balanced nouns denote roles that are not stereotypically associated with either sex and their masculine and feminine forms are used with comparable frequency (e.g., *der Kollege* ‘the.M colleague.M’ – *die Kollegin* ‘the.F colleague.F’). Male-biased nouns denote roles that are stereotypically associated with males and their masculine form is considerably more frequent than the feminine form (e.g., *der Techniker* ‘the.M technician.M’ – *die Technikerin* ‘the.F technician.F’). Zero-plural nouns have identical singular and plural forms, so that number is marked only on the determiner (e.g., *der Lehrer* ‘the.M.SG teacher’ – *die Lehrer* ‘the.PL teacher.PL’) and other elements in morphosyntactic agreement relations. Overt-plural nouns additionally mark plural with a suffix (e.g., *der Journalist* ‘the.M.SG journalist’ – *die Journalisten* ‘the.PL journalist.PL’). Other lexical properties (e.g., frequency, length, imageability) were controlled for and balanced across groups (see Supplementary Tables S1a,b).

All verbs in the sentences appeared in synthetic tense forms (i.e., present or preterite). Particle verbs, function/light verb constructions (*Funktionsverbgefüge*), and auxiliary verbs were avoided. These restrictions were implemented to prevent delayed semantic interpretation of the verb, which can occur in German when parts of the predicate appear only at the end of the sentence. In all experimental sentences, the verb appeared immediately after the subject noun phrase. In addition, sentences did not contain anaphoric or possessive pronouns referring back to the subject. These constraints ensured that all experimental manipulations were confined to the first three words of the sentence. Following the finite verb, the number of arguments and the overall sentence structure varied. The average sentence length was 8.23 words (range: 6–11 words).

To implement the two manipulations, sentence pairs were created around the same role noun. In both manipulations, one member of the pair contained the noun in its masculine singular form (1a, 2a); this base sentence was identical in the gender and the number manipulation. In the gender manipulation, the base sentence was paired with a sentence containing the feminine singular form of the same noun (1b). In the number manipulation, it was paired with a sentence containing the masculine plural form (2b), which also required plural agreement on the verb. Each item thus occurred in four conditions, which were distributed across eight presentation lists according to a Latin square design so that each participant saw each item only once.

Examples of experimental stimuli (ROIs underlined):

‘The photographer makes the most money from wedding photos.’

1) Gender manipulation:

- *Der Fotograf* *verdient das meiste Geld mit Bildern von Hochzeiten*.‘the.M.SG photographer makes.SG…’
- *Die Fotografin* *verdient das meiste Geld mit Bildern von Hochzeiten.*‘the.F.SG photographer.F makes.SG…’

2) Number manipulation:

- *Der Fotograf verdient das meiste Geld mit Bildern von Hochzeiten.*‘the.M.SG photographer makes.SG…’
- *Die Fotografen verdienen das meiste Geld mit Bildern von Hochzeiten.*‘the.M.PL photographers makes.PL…’

Each sentence pair thus formed a minimal contrast in which the subject noun phrase differed only in gender or only in number (the latter entailing corresponding verb agreement).^3^

In addition, 96 sentences served as fillers (item-to-filler ratio = 1:3). The filler items matched the experimental sentences in length and syntactic complexity but covered a wide range of topics and grammatical structures. Care was taken to ensure that no critical lexical material (e.g., the subject nouns and the verbs) from the experimental sentences appeared in the filler sentences. All materials (nouns, sentences) are provided in a public online repository.^4^

### 2.3 Apparatus and procedure

The experiment was conducted using an EyeLink Portable Duo system and programmed with EyeLink Experiment Builder (Version 2.3.38; SR Research Ltd., 2020). Participants’ heads were stabilized using the SR Research Head Support. Eye movements were recorded binocularly at a sampling rate of 500 Hz; analyses were based on data from participants’ dominant eye. Stimuli were presented on a 16-inch laptop display (1,920 × 1,080 px). Sentences appeared in black, monospaced sans-serif font (Consolas, 20 pt) on a light grey background, vertically centered and left-aligned with a 200-pixel (≈4 cm) margin. All sentences were displayed on a single line without line breaks. Prior to each of the four experimental blocks, the eye tracker was calibrated using a 13-point calibration procedure with the standard EyeLink settings. Calibration accuracy was assessed by the experimenter using the EyeLink validation procedure. A drift check was performed before each trial. Participants were instructed to fixate a target presented on the screen, and the experimenter initiated the trial by pressing the space bar once the participant’s gaze was visually judged to be located on the target. If the gaze position was judged to be too far from the target, the participant was recalibrated before the trial continued.

Participants were tested individually and seated approximately 60 cm from the display. After reading the instructions, participants completed a short familiarization phase before beginning the experiment.

The experiment consisted of four blocks of equal length, with breaks in between. Each block contained 48 trials: eight critical sentences analyzed in the present study, eight filler repetitions of critical sentences (identical or modified; analyzed in a separate memory study), and 32 other filler sentences with varied syntactic structures. In total, each participant read 192 sentences, including 32 critical items relevant to the present analyses. Items and conditions were distributed across eight experimental lists using a Latin square design. Each participant was assigned to one list and therefore saw each of the 32 critical items only once, in one of the four possible conditions (gender: eight masculine, eight feminine; number: eight singular, eight plural).

Block order was randomized individually for each participant. Within blocks, item presentation was pseudo-randomized with the following constraints: the first trial of each block was always a filler; no more than three trials with the same critical feature (singular, plural, feminine, masculine) occurred consecutively.

Each trial began with a fixation point near the left margin at the position of the first word of the upcoming sentence. All sentences were preceded by the phrase *Hier steht:* (“This says:”) to ensure that the first fixated word did not belong to the critical subject noun phrase. After reading the sentence, participants moved their gaze to an arrow in the bottom-right corner of the screen; fixating this arrow for at least 300 ms initiated the next trial.

To ensure that participants read each sentence carefully and maintained their attention throughout the experiment, control questions were included after a subset of filler sentences. In 32 trials, once the sentence had disappeared from the screen, participants were asked whether the last sentence already occurred earlier in the experiment. Responses were given via two buttons corresponding to “yes” and “no.” Expected correct answers were balanced (16 yes, 16 no responses). Control questions were evenly distributed, with eight questions per block, and their exact position within each block was pseudo-randomized.

### 2.4 Data handling and analysis

The data addressing the number and gender manipulations were analyzed separately as two sub-experiments, since each manipulation covered critical regions of different length and involved different types of manipulation. All analyses were conducted in R, version 4.5.2, using a reproducible workflow. Preprocessed data and R analysis scripts are provided in a public online repository (OSF link: https://doi.org/10.17605/OSF.IO/3K4WV).

#### 2.4.1 Data quality

Calibration accuracy was evaluated based on the EyeLink validation results obtained before each experimental block. For participants included in the final analyses, validation data were available for 536 of the 556 expected block-level calibrations (four blocks per participant). Across these 536 calibrations, the mean average validation error was 0.37° (*SD* = 0.16°), while the mean maximum validation error was 0.89° (*SD* = 0.48°). Five participants had no usable validation information for any of their four blocks. These missing validation records were not treated as evidence of poor calibration and were not used to infer calibration accuracy.

#### 2.4.2 Data preparation and preprocessing

Fixations and saccades were identified in SR Research’s Data Viewer (Version 5.1.1; SR Research Ltd., 2025) using the software’s standard event-detection algorithm. The built-in four-step data-cleaning procedure was applied with default settings, except that the minimum fixation duration was lowered from 140 ms to 100 ms to ensure that shorter, linguistically meaningful fixations were retained. All eye-tracking data were manually inspected for drifts, track losses, and other artefacts prior to analysis. In addition, one item was removed from analysis because multiple participants reported unfamiliarity with the word, a pattern that was also evident in their reading behavior. Reading measures for the predefined regions of interest (ROIs) were extracted using Data Viewer’s *Get Reading Measures* tool.

Regions of interest (ROIs) were defined based on linguistic criteria. In each case, the ROI encompassed all linguistic material directly affected by the respective feature manipulation. In both experiments, the critical ROIs consisted of the subject noun phrase (determiner + noun). For the number manipulation, the following verb was additionally included, as it carries number agreement morphology and therefore reflects the manipulated feature (see Examples of Experimental Stimuli above). Technically, ROIs were implemented with Data Viewer’s default settings for text with 30-pixel margins to the right and left of the text but with additional 30 pixels (i.e., 60-pixel in total) margins above and beneath the text.^5^

#### 2.4.3 Early and late processing measures

Two processing stages were analyzed. Early processing was indexed by *gaze duration* in the critical region of interest, capturing the first pass through the region. Such early reading measures are considered to reflect initial lexical access and first-pass integration (Palma and Titone, 2020). Late processing was indexed by two measures: (a) the *probability of regressions*, a binary indicator of whether readers initiated a regression into the critical region, and (b) *rereading time*, defined as the sum of all fixations on the critical region that occurred after the reader had first exited it. Such measures are indicative of integrative processing and reanalysis (Roberts and Siyanova-Chanturia, 2013).

Rereading time was analyzed only for trials in which a regression occurred. This conditional approach follows standard practice in eye-tracking research: regression probability and rereading time reflect distinct cognitive processes (Clifton et al., 2007; Schotter and Dillon, 2025). Regression probability captures the initiation of reanalysis—whether readers detect a mismatch, uncertainty, or processing difficulty that prompts them to move their eyes back to earlier material. In contrast, rereading time reflects the depth and cost of reanalysis, indexing how long readers spend resolving the difficulty once they have committed to re-examining the region.

#### 2.4.4 Statistical modelling

All mixed-effects models included *Language* (L1 vs. L2), the relevant manipulation (gender: *Balancedness* yes/no; number: *Plural Morpheme* overt/zero), and *Form* (gender: feminine/masculine; number: singular/plural) as fixed effects, along with all interactions. Following recommendations for maximal random-effects structures (Barr et al., 2013), we initially attempted to fit models including random slopes for participants and items. However, these models showed severe convergence issues and overparameterization, consistent with concerns raised by Bates et al. (2015) and Matuschek et al. (2017), so we retained random-intercept–only models.

Gaze duration and rereading time were analyzed using Gamma generalized linear mixed-effects models with a *log* link, implemented in *glmmTMB* (Brooks et al., 2017), which provides flexible and numerically stable estimation for Gamma and binomial GLMMs with complex interaction structures. Gamma GLMMs are well suited for reading-time data, which are strictly positive and typically exhibit strong right-skew (Kliegl et al., 2006; Staub and Benatar, 2013).

Regression probability was analyzed using binomial mixed-effects models with a *logit* link (Jaeger, 2008). The dependent variable was a binary trial-level indicator: trials containing at least one regression targeting a word within the critical ROI were coded as 1, and trials without such a regression as 0. Model-based predicted probabilities were obtained by applying the inverse-*logit* transformation to the fitted values.

For all models, Type II Wald χ^2^ tests were used to assess the significance of main effects and interactions. Model-based estimates and pairwise comparisons were obtained using *emmeans* (Lenth and Piaskowski, 2026). Detailed results of all models (significance tests) are provided in the Supplementary Material (see also public repository for all R analysis scripts and outputs).

## 3 Gender experiment

### 3.1 Results

#### 3.1.1 Gaze duration

The gaze-duration results for the gender experiment are visualized in Figure 1.

**Figure 1:**
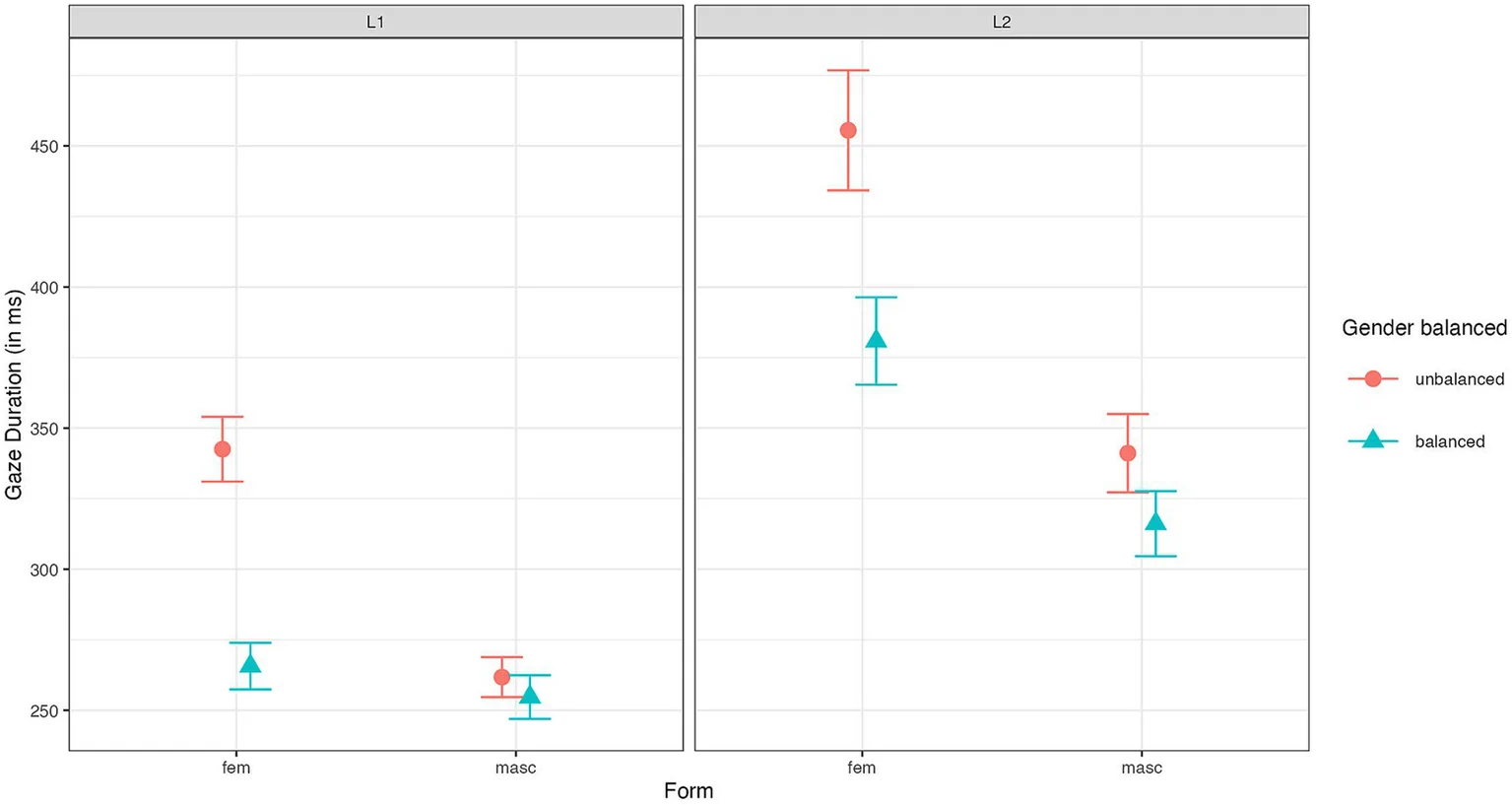
Mean gaze durations in the gender experiment with standard errors.

A Gamma mixed-effects model with a *log* link revealed main effects of *Language*, *Balancedness*, and *Form*. Gaze durations were longer in L2 than in L1, χ^2^(1) = 31.90, *p* < 0.001, longer for unbalanced than balanced nouns, χ^2^(1) = 6.30, *p* = 0.012, and longer for feminine than masculine forms, χ^2^(1) = 104.79, *p* < 0.001. Two interactions reached significance. The *Language* × *Form* interaction, χ^2^(1) = 5.27, *p* = 0.022, reflected a larger feminine–masculine difference in L2 (ratio = 1.26) than in L1 (ratio = 1.16), indicating higher costs for the suffixed feminine form in L2. The *Balancedness* × *Form* interaction, χ^2^(1) = 16.57, *p* < 0.001, reflected a larger balanced–unbalanced difference for feminine than for masculine nouns. Specifically, feminine forms of male-biased nouns (unbalanced, e.g., *die Technikerin* ‘the.F technician.F’) showed longer fixations than feminine forms of balanced nouns (*die Lehrerin* ‘the.F teacher.F’) (ratios = 1.29 in L1; 1.19 in L2). Masculine forms showed little difference between balanced and unbalanced nouns (ratios ≈ 1.04–1.08). A small three-way interaction did not reach significance, χ^2^(1) = 2.88, *p* = 0.090, but indicated a tendency for larger *Balancedness* effects for feminine nouns in L1 than in L2 (see Figure 1).

Pairwise contrasts confirmed that the balanced vs. unbalanced contrast was significant for feminine forms in L1 (*z* = 3.98, *p* < 0.001) and in L2 (*z* = 2.76, *p* = 0.006), but not for masculine nouns (L1: *z* = 0.55, *p* = 0.585; L2: *z* = 1.27, *p* = 0.203).

Together, findings indicate that a conceptual–lexical conflict of gender information modulates early processing, with longer reading times for unbalanced feminine nouns (i.e., feminine nouns with a masculine gender bias) in both L1 and L2 German.

#### 3.1.2 Regression probability

The regression-probability results for the gender experiment are presented in Figure 2.

**Figure 2:**
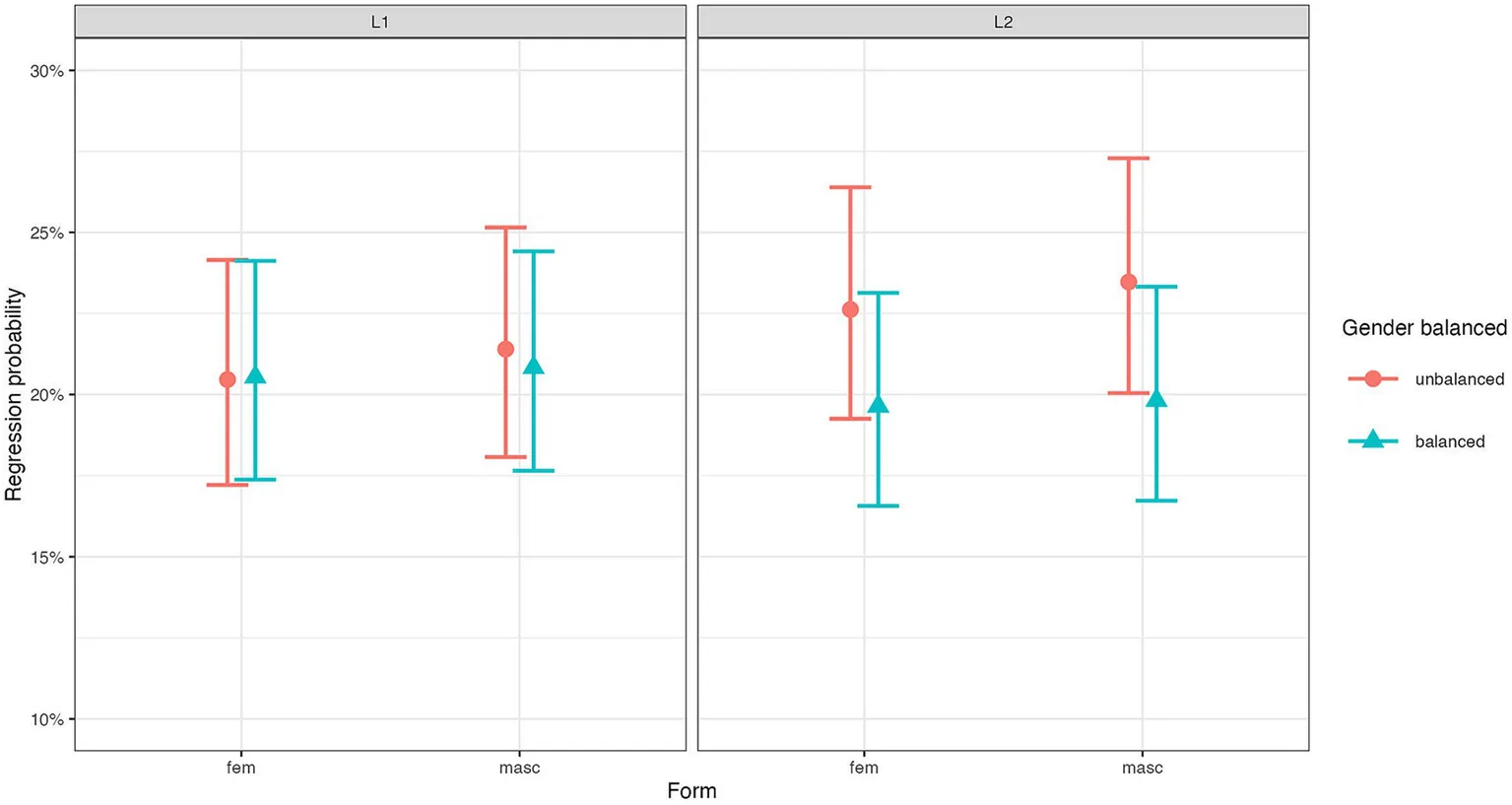
Regression probability into critical ROIs in the gender experiment with standard errors.

A binomial mixed-effects model showed no significant main effects (*Language* χ^2^(1) = 0.29, *p* = 0.592; *Balancedness* χ^2^(1) = 1.84, *p* = 0.175; *Form*, χ^2^(1) = 0.11, *p* = 0.736) and no significant interactions (all χ^2^ ≤ 1.70, all *p* ≥ 0.19). Thus, the conceptual–lexical conflict did not affect the likelihood of initiating reanalysis (see Figure 2).

#### 3.1.3 Rereading time

The rereading-time results for the gender experiment are visualized in Figure 3.

**Figure 3:**
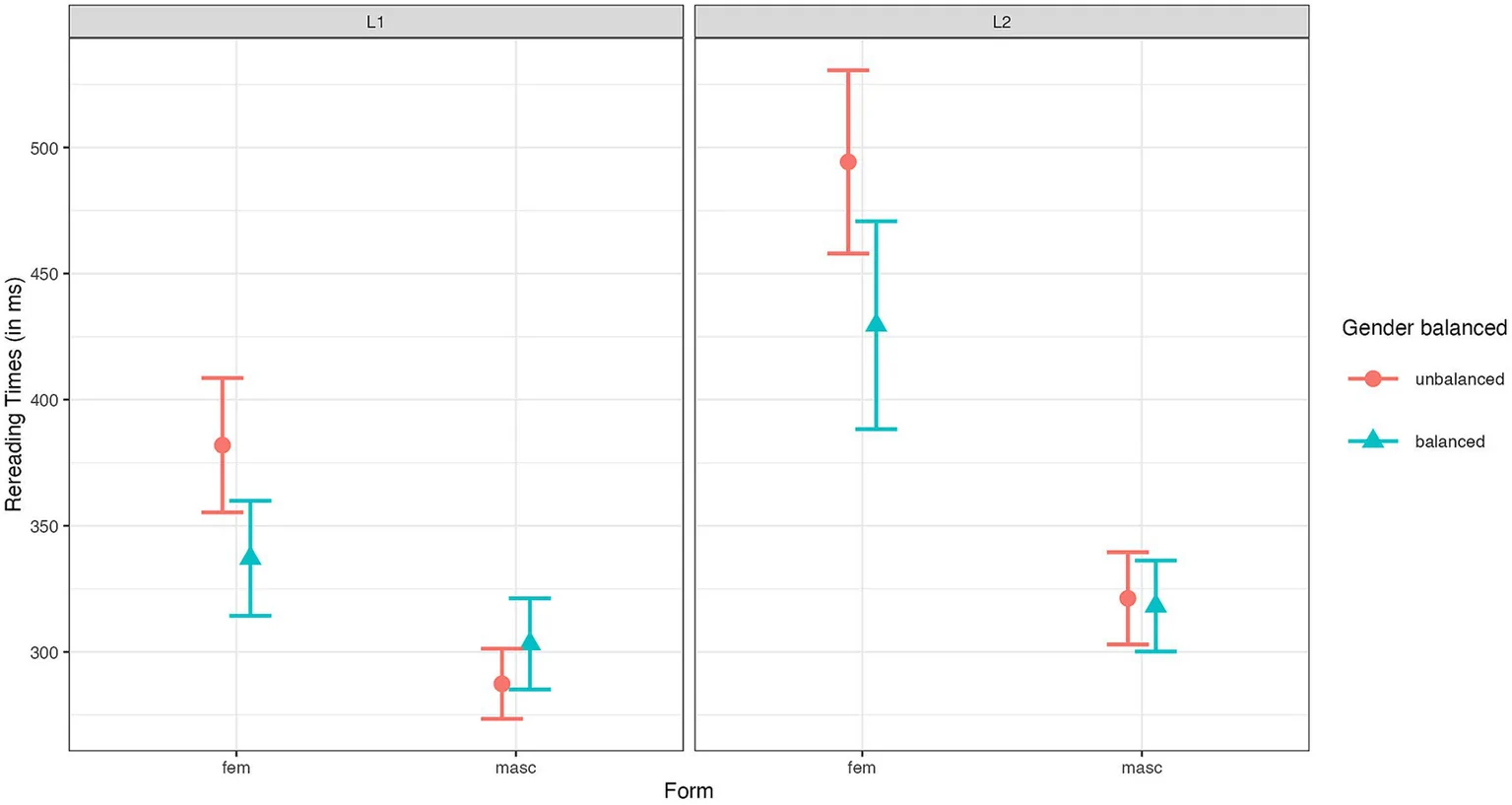
Mean rereading times in the gender experiment with standard errors.

Rereading-time analyses showed main effects of *Language*, χ^2^(1) = 6.11, *p* = 0.013 and *Form*, χ^2^(1) = 54.75, *p* < 0.001, plus significant *Language* × *Form*, χ^2^(1) = 4.50, *p* = 0.034, and *Balancedness* × *Form* interactions, χ^2^(1) = 5.91, *p* = 0.015. Mirroring gaze duration, rereading (when regressions occurred) was longer for feminine forms of male-biased nouns, with no corresponding effects for masculine nouns (see Figure 3).

### 3.2 Discussion

The gender experiment showed a consistent pattern across processing stages. In early measures, feminine forms of male-biased nouns elicited longer fixations than feminine forms of balanced nouns. Feminine forms were also read longer overall, with a larger feminine–masculine difference in L2 than in L1, indicating that L2 readers might have more challenges when processing morphologically complex words. Exploratory analyses of the L2 data including LexTALE scores showed that proficiency modulated this difference in gaze duration (LexTALE × *Form*, χ^2^(1) = 5.01, *p* = 0.025) but not the effect of gender bias (LexTALE × *Balancedness* × *Form*, χ^2^(1) = 1.00, *p* = 0.319). In regression probability and rereading time, proficiency did not interact with any of the manipulations (all *p* ≥ 0.177; see the analysis scripts in the public repository). These effects point to early lexical processing costs rather than structural integration problems.

Late measures provided no evidence that gender or gender bias triggered reanalysis: regression probabilities were uniform (≈20%) and unaffected by the manipulations. When regressions did occur, rereading times mirrored the gaze-duration pattern.

Overall, the gender experiment demonstrates that gender information—and especially conflicting gender bias in feminine forms—shapes early lexical processing, while we do not find evidence that it would trigger late-stage reanalysis. Resolving the lexical-conceptual conflict thus seems to be restricted primarily to lexical access, without major consequences for later processing and without differences between L1 and L2.

## 4 Number experiment

### 4.1 Results

#### 4.1.1 Gaze duration

The gaze-duration results for the number experiment are presented in Figure 4.

**Figure 4:**
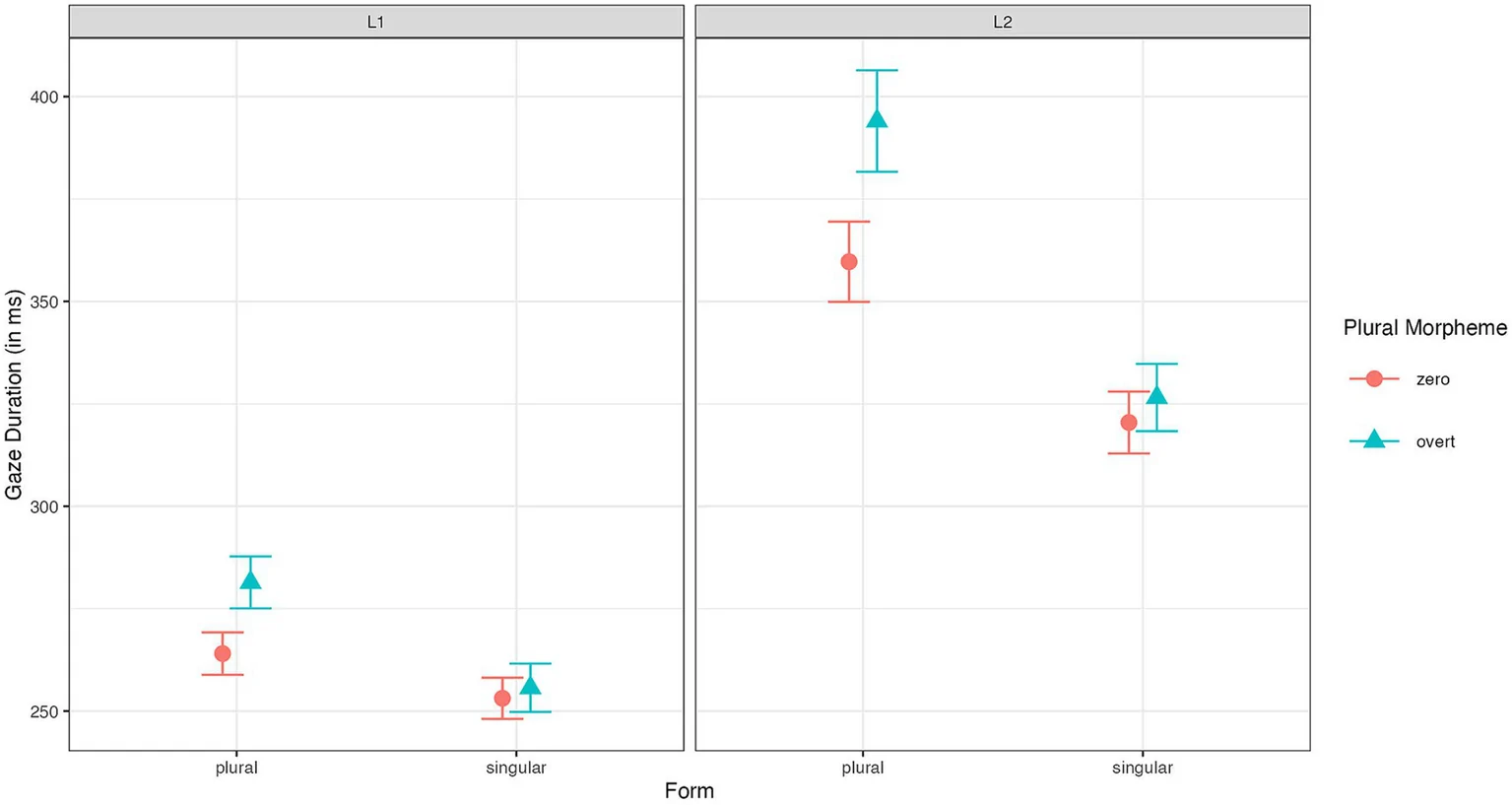
Mean gaze durations in the number experiment with standard errors.

Gaze durations showed main effects of *Language* (χ^2^(1) = 57.5, *p* < 0.001) and *Form* (χ^2^(1) = 59.3, *p* < 0.001): L2 readers had longer gaze durations than L1 readers, and plural forms were read longer than singular forms.

Two interactions were significant. *Language* × *Form*, χ^2^(1) = 6.44, *p* = 0.011, showed a larger plural–singular difference in L2 (estimate = 0.133, *z* = 7.45, *p* < 0.001) than in L1 (estimate = 0.067, *z* = 3.58, *p* < 0.001), indicating, similar to the gender experiment, that processing of morphologically complex forms may be more challenging for L2 readers. The *Plural Morpheme* × *Form* interaction, χ^2^(1) = 7.47, *p* = 0.006, showed that the increase in reading times in plural compared to singular was larger when an overt morpheme was present, consistent with a length/morphological complexity effect rather than conflict-specific early processing. Overall, early reading times in the number experiment were driven by general surface properties such as word length and morphological complexity (i.e., longer for plural forms and overt plural morphology) rather than by a conflict-like pattern associated with number marking (see Figure 4).

#### 4.1.2 Regression probability

The regression-probability results for the number experiment are visualized in Figure 5.

**Figure 5:**
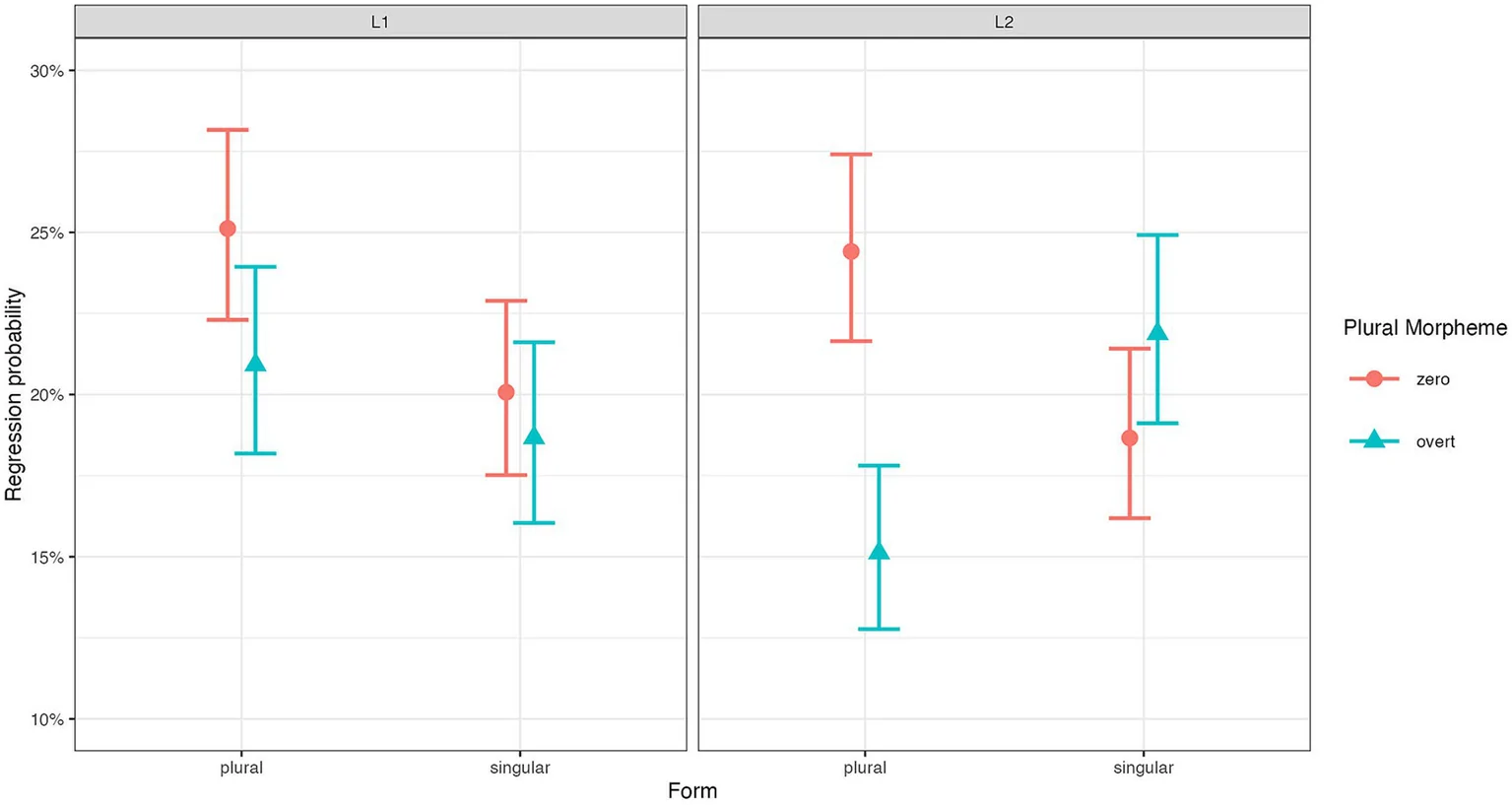
Mean regression probability in the number experiment with standard errors.

Regression probability showed a main effect of *Plural Morpheme* (higher for zero plurals), χ^2^(1) = 4.92, *p* = 0.027, and a marginal effect of *Form* (more regressions to plurals), χ^2^(1) = 2.99, *p* = 0.084. Two interactions clarified the pattern. *Language* × *Form*, χ^2^(1) = 4.12, *p* = 0.042, indicated that plural forms increased regression probability in L1 (estimate = 0.234, *z* = 2.52, *p* = 0.012) but not in L2 (estimate = −0.053, *z* = −0.57, *p* = 0.569). *Plural Morpheme* × *Form*, χ^2^(1) = 12.23, *p* < 0.001, revealed that plural forms with zero plural morphemes elicited more regressions than plural forms with an overt plural morpheme (estimate = 0.490, *z* = 3.80, *p* < 0.001), while singular forms were unaffected (estimate = −0.007, *z* = −0.05, *p* = 0.959). These two interactions jointly produced a pattern that visually resembles a three-way interaction, with the largest numerical difference between plural forms with zero and overt plural morphemes in L2 (see Figure 5), although the *Language* × *Plural Morpheme* × *Form* interaction did not reach the traditional significance level, χ^2^(1) = 2.53, *p* = 0.112 (see Figure 5).

To further investigate the overt vs. zero contrast across number contexts, we computed an interaction contrast. Supporting the visual pattern shown in Figure 5, the *Plural Morpheme* × *Form* contrast was reliable in L2 (odds ratio = 2.051, *z* = 3.60, *p* < 0.001), indicating higher regression probability for zero- than overt-marked plural forms in plural, but not singular, contexts. In L1, the same interaction contrast was not significant (odds ratio = 1.315, *z* = 1.39, *p* = 0.165).

Overall, regressions increased specifically when plural forms lacked an overt plural morpheme, with the clearest effect in L2 readers. The results suggest that L2 readers rely more strongly on overt morphological cues and that the formal–structural conflict in zero plurals—in contrast to the conceptual–lexical gender conflict—is not resolved locally but propagates and also affects later stages of sentence processing.

#### 4.1.3 Rereading time

The rereading-time results for the number experiment are presented in Figure 6.

**Figure 6:**
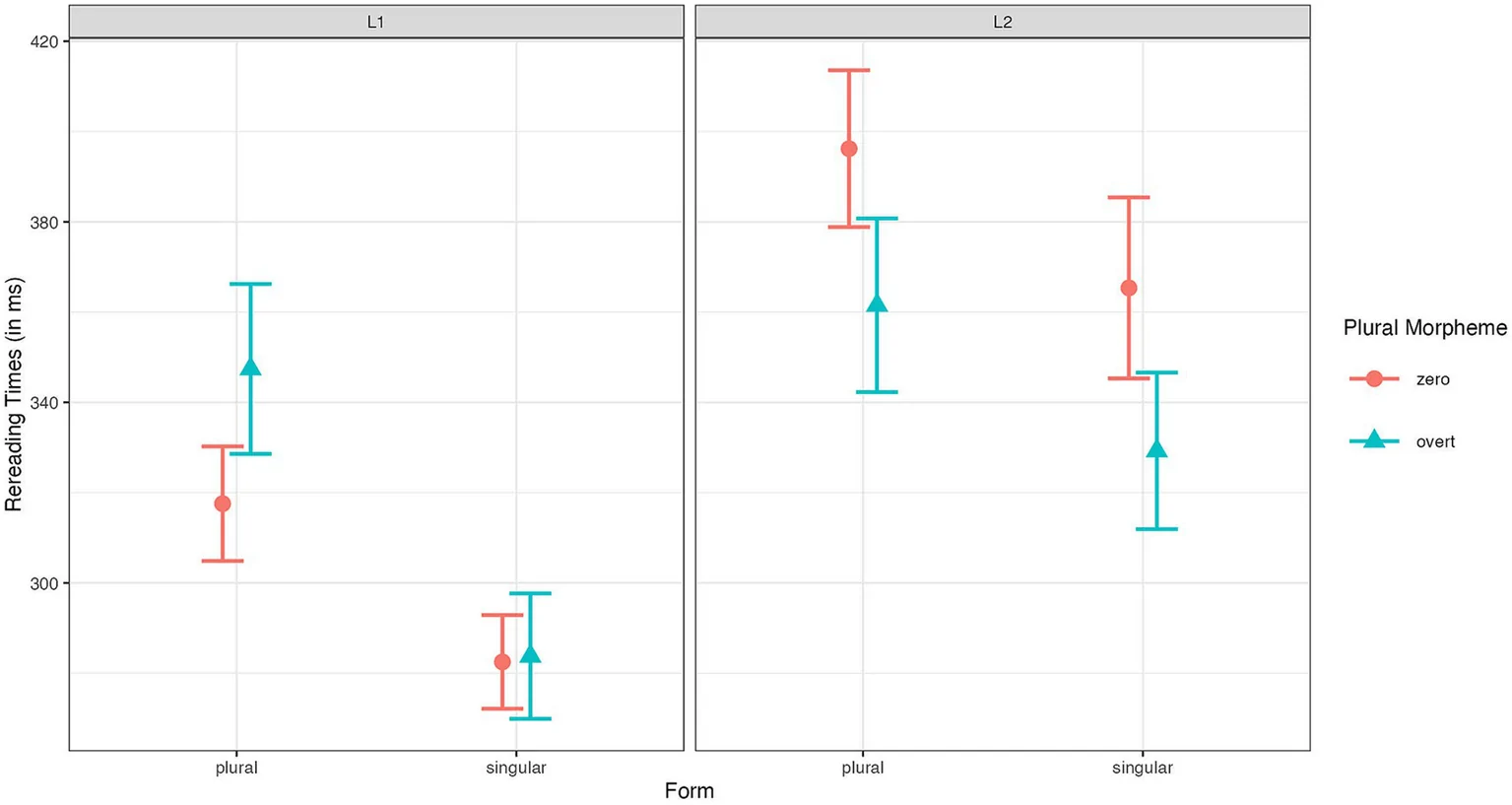
Mean rereading times in the number experiment with standard errors.

Rereading-time analyses showed main effects of *Language*, χ^2^(1) = 10.35, *p* = 0.001, and *Form*, χ^2^(1) = 22.97, *p* < 0.001, indicating that L2 readers showed generally longer rereading times than L1 readers, and that plural forms elicited longer rereading times than singular forms. Crucially, the significant *Language* × *Plural Morpheme* interaction, χ^2^(1) = 4.08, *p* = 0.043, showed that rereading took longer for number-ambiguous forms in L2 (*z* = 1.86, *p* = 0.062), but not in L1 (*z* = −0.39, *p* = 0.700) (see Figure 6).

Taken together, rereading did not mirror the regression-probability effects. In L1, rereading resembled first-pass processing (longer for plurals, numerically strongest for overt plurals). In L2, rereading was generally longer for zero-plural nouns, suggesting that once L2 readers regressed, they were occupied with number assignment of these forms in both singular and plural contexts.

### 4.2 Additional exploratory analyses

Because the number experiment showed clear L1–L2 differences, we ran exploratory follow-up analyses to better characterize these group contrasts, especially in late measures. For detailed statistical results of these additional analyses, see the Supplementary Material and the R script in the public repository.

#### 4.2.1 Regression probability by sub-ROI (determiner – noun – verb)

We examined regression probabilities for plural contexts by target location within the agreement domain (sub-ROIs: determiner, noun, verb). The main analysis suggested higher reanalysis rates for morphologically ambiguous plurals [e.g., *der Lehrer* (singular) – *die Lehrer* (plural)], especially in L2. Splitting the critical region by sub-ROIs may shed light on which cues contribute to what degree to resolving the formal–structural conflict in plural contexts. Given the relatively small number of regressions distributed across the three sub-regions, statistical significance was unlikely, but the analyses may help identify promising directions for future research.

The sub-ROI regression-probability patterns in plural contexts are presented in Figure 7.

**Figure 7:**
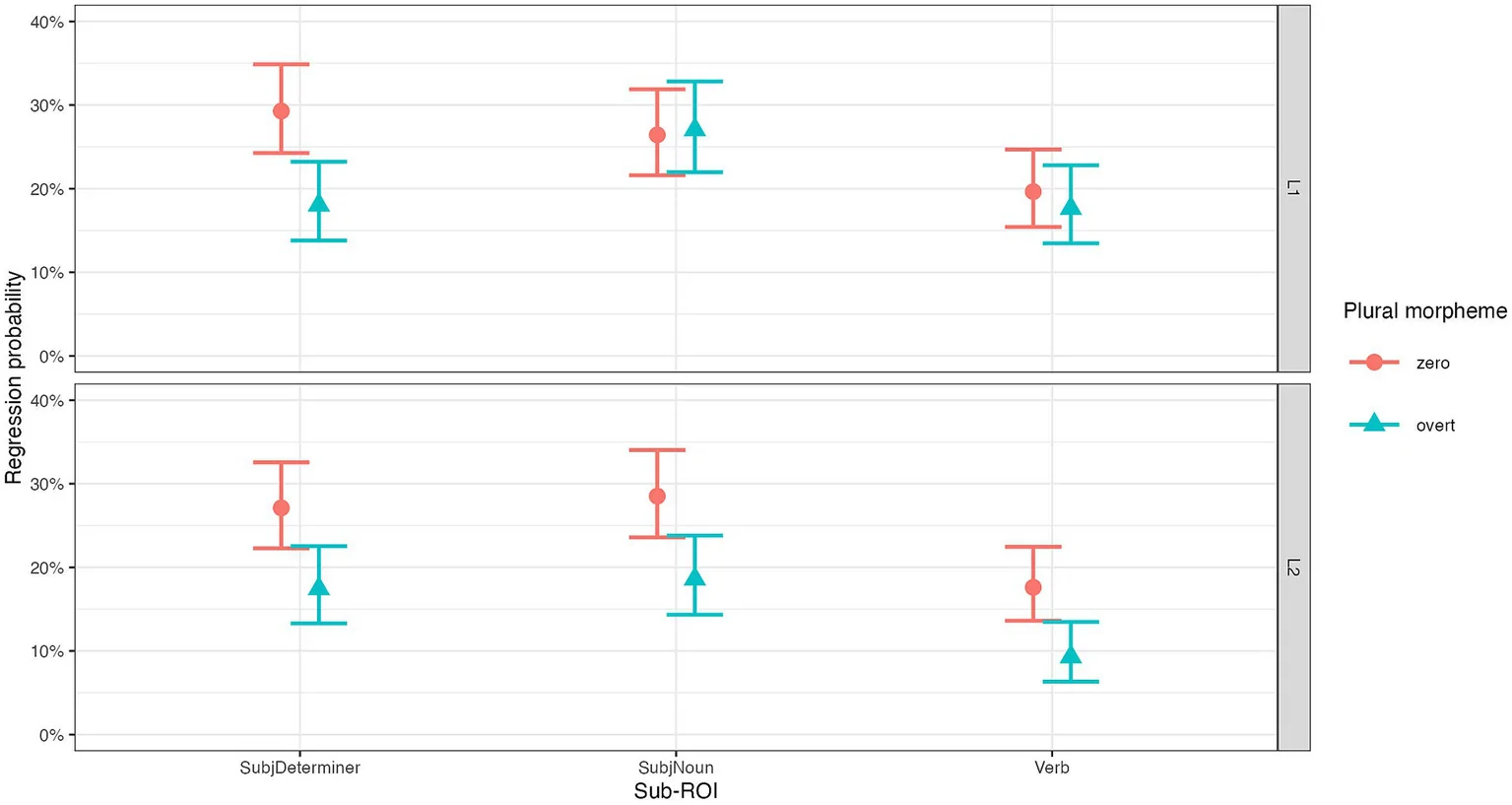
Regression probability in plural contexts in the number experiment by sub-ROI with standard errors.

Descriptively, L2 readers showed higher regression probability for zero plurals across all three sub-ROIs, whereas L1 readers showed an increase mainly to the determiner. This suggests that L1 readers relied primarily on determiner marking for disambiguation, while L2 readers searched for cues across the entire agreement region.

A full model including *ROI* as an additional fixed effect (*Language* × *Form* × *Plural Morpheme* × *ROI*) was underpowered and yielded no significant higher-order interactions (e.g., *Language* × *Plural Morpheme* × *Form* × *ROI*: χ^2^(2) = 2.09, *p* = 0.350). We therefore ran separate exploratory analyses for L1 and L2 in plural contexts only, with fixed effects for sub-ROI and *Plural Morpheme*.

In L1, *ROI* × *Plural Morpheme* was significant, χ^2^(2) = 6.474, *p* = 0.039. Pairwise contrasts showed that the effect of *Plural Morpheme* was significant only at the determiner (estimate = 0.753, *z* = 2.96, *p* = 0.003), with higher regression probability for the zero-plural condition.

In L2, the interaction was not significant, χ^2^(2) = 0.228, *p* = 0.892, but there was a robust main effect of *Plural Morpheme*, χ^2^(1) = 13.425, *p* < 0.001. The zero-plural increase was present at all sub-ROIs: determiner (estimate = 0.618, *z* = 2.46, *p* = 0.014), noun (estimate = 0.613, *z* = 2.48, *p* = 0.013), and verb (estimate = 0.766, *z* = 2.57, *p* = 0.010).

#### 4.2.2 Within-L2 comparisons: proficiency level

We next asked whether L1–L2 differences reflect a categorical native/non-native distinction or are modulated by L2 proficiency. The L2 group included B2 (*n* = 20) and C1 learners (*n* = 50) and spanned 66.25–96.25% on the modified LexTALE test (*M* = 81.85%, *SD* = 7.40). We focused on regression probability and rereading time, the measures showing clearest group differences.

Adding LexTALE to the main regression-probability model yielded no significant interactions (all *p* > 0.430). However, when LexTALE was included as a fixed effect in the analysis that also included the sub-ROIs for plural forms, a significant three-way interaction emerged (LexTALE × *Plural Morpheme* × *ROI*), χ^2^(2) = 6.03, *p* = 0.049 (see Figure 8).

**Figure 8:**
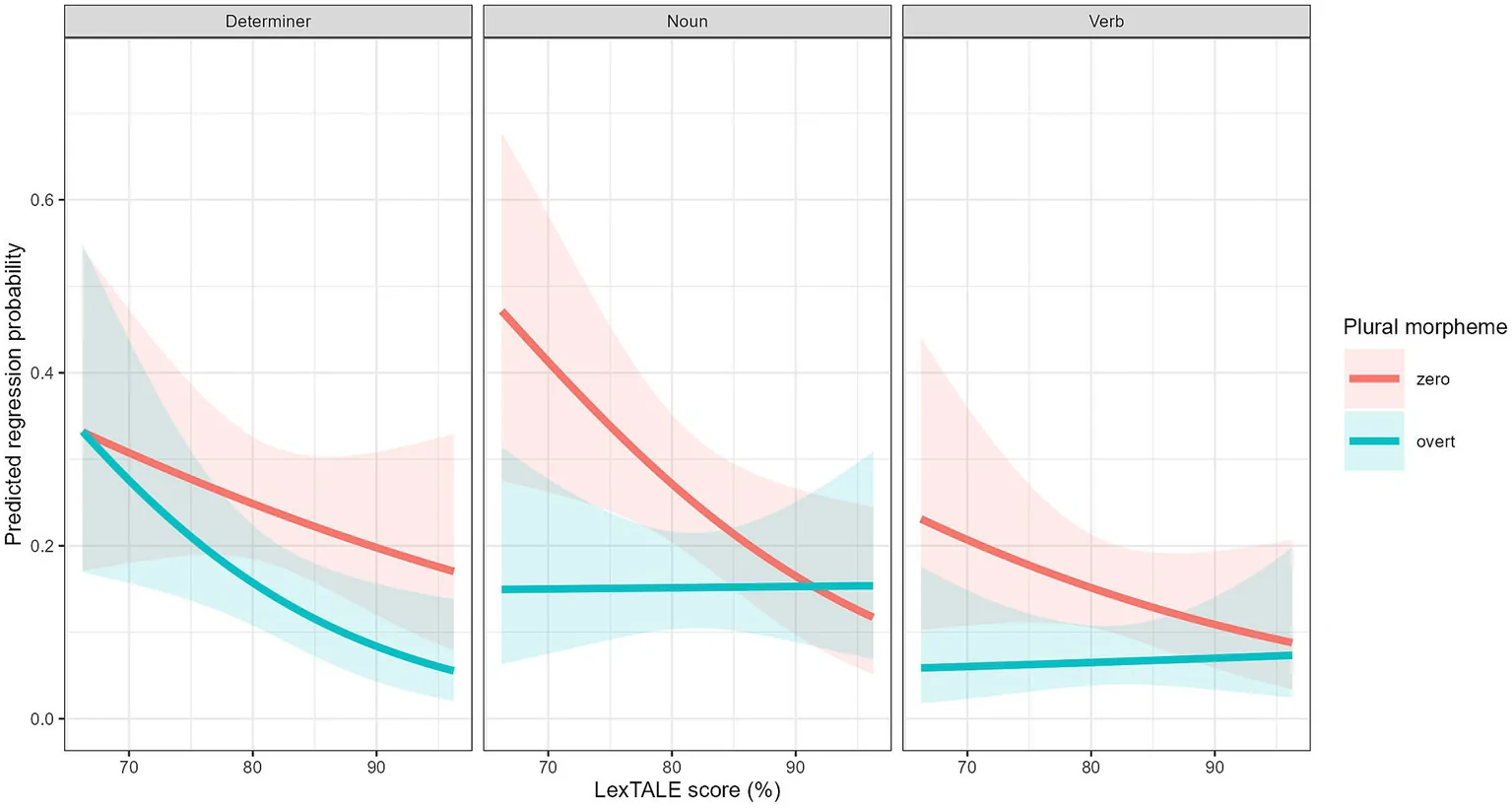
Model-based predictions of regression probability in plural contexts in L2 readers by proficiency, plural morpheme, and sub-ROI with 95% confidence intervals.

The proficiency-related regression-probability patterns are visualized in Figure 8.

As suggested by Figure 8, the difference in regression probabilities between overt- and zero-marked plurals changes with increasing proficiency (x-axis), and, interestingly, the nature of this change is different for the three sub-ROIs. For noun and verb, the difference is larger at lower LexTALE scores and decreases with higher scores. On the determiner, in contrast, there is no reliable difference between overt- and zero-marked plurals for less proficient L2 readers, whereas for more proficient readers, regression probabilities are higher for zero-marked plural forms than for overt forms. Thus, the more advanced participants in our study show a pattern more similar to the L1 participants: zero-marked plural forms trigger more regressions to the determiner and fewer to the noun and verb.

The main analysis for rereading times (see above) revealed longer rereading times for zero-plural role nouns than for overt-plural role nouns both in singular and in plural in L2, while no such differences were observed in L1. An additional exploratory analysis for L2 readers with their LexTALE score added to the model showed that proficiency modified this effect: LexTALE × *Plural Morpheme*, χ^2^(1) = 7.65, *p* = 0.006. As indicated in Figure 9, the L2 effect of longer rereading times for zero plurals was mainly driven by participants with lower LexTALE scores.

**Figure 9:**
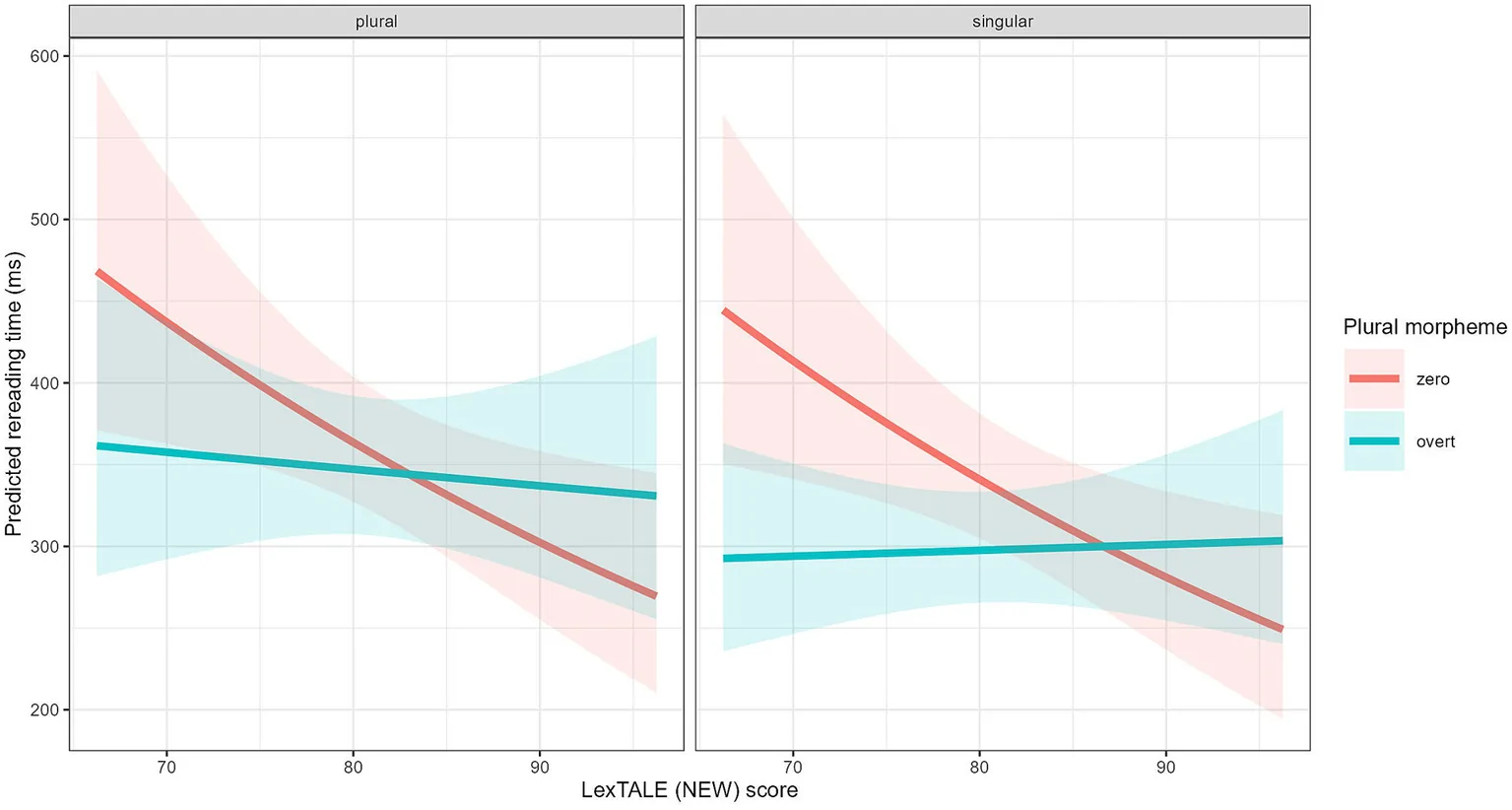
Model-based predictions of rereading time in L2 readers by proficiency and plural morpheme with 95% confidence intervals.

### 4.3 Discussion

The number experiment revealed a clear dissociation between early lexical processing and later morphosyntactic integration, and between L1 and L2 readers. In first pass, both groups showed longer gaze durations for plural forms with an overt plural morpheme, indicating that first-pass processing is primarily driven by surface properties such as word length and morphological complexity.

Late measures instead reflected integration difficulty. Regression probability increased for zero-marked plurals in plural contexts, most clearly in L2 readers, suggesting that L2 comprehenders initiate reanalysis when an expected overt cue is absent and rely more on explicit morphological marking during agreement computation. The exploratory analysis specified that L1 readers primarily use determiner marking to disambiguate zero plurals, whereas L2 readers search for number cues across determiner, noun, and verb.

Rereading also showed a *Language* × *Plural Morpheme* interaction. In L2, reanalysis of morphologically ambiguous forms (e.g., *der Lehrer* (singular) – *die Lehrer* (plural)) incurred prolonged rereading in both plural and singular contexts, indicating that zero morphology is a general bottleneck during reanalysis. Exploratory analysis further showed that the effect was primarily driven by less advanced learners. L1 readers showed no comparable difficulty with morphologically ambiguous forms; their rereading largely mirrored gaze duration, similarly like the rereading behavior of highly advanced L2 readers in the exploratory analysis.

## 5 General discussion

The present eye-tracking study tested how readers resolve two sources of processing difficulty during German sentence reading: a conceptual–lexical conflict between stereotypical gender expectations and explicit gender morphology on role nouns, and a formal–structural conflict associated with zero-marked plural nouns. Because zero plurals are formally identical to their singular forms, number must be inferred from agreement cues elsewhere in the sentence. By comparing L1 and L2 readers within the same design, the study isolates how conceptual knowledge and morphosyntactic cues contribute to comprehension across processing domains and learner groups. Across experiments, conceptual–lexical conflict (gender bias) affected early lexical access and was resolved locally in both L1 and L2, with no clear consequences for later processing. In contrast, the formal–structural number conflict (zero plurals) emerged primarily in later measures, especially in L2 readers, indicating protracted agreement integration and reanalysis when overt number cues are absent. This domain-specific L1/L2 dissociation contributes to models of bilingual sentence processing.

The gender experiment demonstrated that stereotype-based expectations influence the processing of role nouns at the earliest stages of lexical access. In the early gaze-duration measure, feminine forms of masculine-biased role nouns elicited longer fixation times than feminine forms of gender-balanced role nouns, while the gaze duration on the masculine forms of these two groups of nouns did not differ. This pattern indicates that readers experience increased processing cost when explicit feminine morphology conflicts with the stereotypical gender associated with the role noun. Crucially, the present study provides the first direct evidence that this cost is triggered specifically at the feminine form of the role noun itself. The fact that this effect emerged immediately at the role noun NP is consistent with previous L1 findings showing that stereotypical knowledge associated with social roles is activated rapidly and automatically during comprehension (Banaji and Hardin, 1996; Carreiras et al., 1996; Garnham et al., 2002; Gygax et al., 2021). Such findings have often been interpreted within memory-based approaches to comprehension, according to which lexical input such as a role noun can trigger the activation of associated conceptual information from long-term memory (e.g., Myers and O’Brien, 1998; Cook and O’Brien, 2014). The present study extends these findings by demonstrating such effects directly and specifically at the feminine form of the role noun, while much prior research has examined stereotype conflict triggered by masculine generic forms and their effects on downstream interpretation (e.g., slower processing of female continuations after masculine generics; Henley and Abueg, 2003; Schmitz et al., 2023).

Our results further show that the stereotype information is actively processed even when not explicitly triggered by elements later in the sentence and that the conflict between gender bias and morphological marking does not affect the frequency of regressions associated with sentence-level reanalysis. This processing profile suggests that the observed difficulty reflects increased lexical processing load rather than structural revision of the sentence representation. In other words, readers appear to resolve the stereotype–morphology mismatch locally during lexical access without triggering broader syntactic or discourse-level repair processes.

This pattern aligns closely with ERP evidence on gender-stereotype violations. In a systematic review of 17 studies, Porkert et al. (2024) demonstrate a clear dissociation: when stereotype information does not serve as a cue for anaphora resolution or sentence-level coherence (as in the present design, where the conflict is confined to the role-noun NP itself), violations reliably elicit an N400 reflecting semantic memory retrieval and integration at the word or local sentence-context level rather than a P600. The P600, by contrast, emerges only when the same stereotype information is required to link past and current information across the sentence (e.g., pronoun resolution or speaker-identity construction), indexing late reanalysis and memory-trace revision (see also Tanner et al., 2017). Porkert et al. interpret this trade-off within multistream and conflict-monitoring frameworks (Van Herten et al., 2006; Kuperberg, 2007), in which the N400 indexes semantic memory processing while the P600 reflects monitoring for perception errors that necessitate structural repair. The present eye-tracking data provide the first direct cross-method parallel to this ERP dissociation. Our early gaze-duration cost (lexical access) with no increase in regressions mirrors the N400 pattern for purely semantic/conceptual mismatches that can be accommodated locally, while the absence of later effects mirrors the lack of P600 when no sentence-wide revision is required.

Crucially, the same early, local resolution was observed in L1 and L2 readers. This similarity indicates that the underlying source of the effect is not primarily linguistic but conceptual in nature. Gender stereotypes associated with social roles are part of general world knowledge and can therefore be shared across languages. As a result, the conceptual expectations activated by a role noun appear to influence lexical processing in a comparable way in both native and non-native language comprehension.

Of particular importance is the fact that we did not observe any indications of decreased sensitivity to gender stereotypes in the L2: The gender-bias-based conceptual–lexical conflict was equally strong in both L1 and L2 readers. This contrasts with recent findings by Jankowiak et al. (2025), who reported evidence (albeit only for a subgroup of participants) suggesting that unbalanced bilinguals may exhibit reduced stereotypical behavior when using their L2. However, in that study, the distinction between L1 and L2 was confounded with a typological contrast between a grammatical gender language (L1 Polish) and a natural gender language (L2 English). It is therefore possible that the reduced sensitivity to gender stereotypes which the authors observed reflected the structural properties of the languages (weaker gender-based effects in a language where gender is less systematically encoded) rather than L2 status per se. In the present study, the L2 participants had Slavic L1s, with Czech speakers forming the largest subgroup, and these L1s share with German the grammatical distinction between masculine, feminine, and neuter gender categories. Our study shows that when the L2 status is examined in a context where the relevant categories are broadly comparable between L1 and L2, stereotype effects can remain completely intact in L2. As Czech speakers formed the majority of the L2 group, these findings apply primarily to advanced learners with Slavic L1s and do not allow separate conclusions about the three L1 subgroups. Moreover, participants’ knowledge of further gender-marked languages was not controlled, so a contribution of these languages cannot be ruled out entirely.

The number experiment addressed a conflict of a fundamentally different kind. As set out in the Introduction, the number manipulation was designed such that the ambiguity would be exclusively formal: plural role nouns referred to multiple referents and agreement was grammatically correct throughout, so that the conceptual number of the referent and its grammatical realization never diverged (cf. Bock and Miller, 1991; Eberhard et al., 2005). The difficulty induced by zero plurals therefore does not arise from the conceptual representation of plurality but from the morphological form, which is uninformative about the number of the referent. This processing profile emerged clearly in the eye-movement data. When plural nouns lack overt plural morphology and are therefore formally identical to their singular counterparts, number interpretation cannot be determined from the noun itself and must instead be inferred from agreement cues elsewhere in the sentence. If the subject of the sentence was a zero plural, readers were more likely to initiate regressions during sentence processing. Unlike the stereotype-driven effects observed in the gender experiment, these integration difficulties showed clear differences between L1 and L2 readers. Although early gaze durations were similar across groups and primarily reflected surface properties such as word length and morphological complexity, later measures revealed that morphologically ambiguous plural forms triggered substantially more reanalysis in L2 readers than in L1 readers. This finding is consistent with cue-based models of agreement processing (Wagers et al., 2009), which posit that underspecified or absent morphological cues on the head noun force reliance on retrieval and integration of number features from distant agreement sites. This leads to greater reanalysis costs (indexed by regressions) when cues are weak or non-local. The amplified L2 reanalysis effect aligns with evidence that non-native comprehenders are particularly sensitive to such morphosyntactic ambiguity in German (see also Lago and Felser, 2018).

The additional exploratory regression analyses offer further novel insight into the precise locus of resolution. For L1 readers, increased regressions associated with zero plurals were localized almost exclusively at the determiner. Because the determiner carries overt number marking, it provides a reliable cue for resolving the ambiguity created by the noun. This localized regression pattern suggests that L1 readers quickly identified and used the determiner as the critical source of information for disambiguating the noun phrase.

L2 readers, by contrast, showed a more distributed pattern. Regression probabilities increased across all subregions of the agreement domain, including the determiner, noun, and verb. Rather than targeting a single diagnostic cue, L2 readers appeared to engage in a broader search for number cues during reanalysis. This pattern indicates that morphologically ambiguous forms triggered a more global reanalysis strategy during L2 sentence processing. The finding is consistent with the Shallow Structure Hypothesis (Clahsen and Felser, 2006), which proposes that L2 processing builds shallower syntactic representations and relies more heavily on surface-level cues. This may result in increased integration costs and distributed regressions when a single diagnostic cue (here, overt plural morphology on the noun) is absent. The rereading-time analyses reinforce this interpretation, revealing increased rereading costs for morphologically ambiguous forms in L2, but not L1 readers.

Importantly, the exploratory proficiency analyses revealed that the L2-specific patterns were primarily driven by the less proficient L2 speakers (roughly at B2 level), while the reading behavior of more advanced readers resembled more that of native speakers. This proficiency modulation was specific to the number experiment: in the gender experiment, proficiency affected only the general processing cost of the suffixed feminine forms, but not the effect of gender bias, in line with the absence of L1–L2 differences for the conceptual–lexical conflict. The proficiency modulation in the number experiment aligns with evidence that highly proficient learners can attain native-like efficiency in cue integration and reanalysis (Hopp, 2010). It also parallels eye-tracking findings showing that intermediate learners of German engage in extensive rereading during morphosyntactic disambiguation, despite being sensitive to local cues such as case marking on the determiner (Jackson et al., 2012).

The asymmetry between the two groups can be related to the way number is distributed across the sentence. Number is a feature of the subject noun phrase (Ritter, 1988, 1993) and is expressed within it on both the noun and the determiner, whereas the verb reflects it only through subject–verb agreement. In zero-marked plurals, the exponent on the lexical head is missing, so that the determiner remains the only phrase-internal cue to number. L1 readers appear to exploit precisely this: they returned to the determiner rather than to the uninformative noun. Less proficient L2 readers, in contrast, regressed across the whole agreement domain, including the verb, and reread ambiguous forms longer, suggesting that they were less efficient in compensating for the missing marking on the noun by means of agreement information. This interpretation converges with acquisition research on the role of overt morphology. In L1 acquisition, meanings that are marked overtly and transparently are acquired earlier than meanings that must be inferred from indirect cues (Slobin, 1973; Van Hout, 1998, 2008), and children even express individual meaning components by separate forms where the adult language uses a single one (Guasti et al., 2023). In L2 acquisition, cues that are low in salience and redundant, i.e., whose meaning is also expressed elsewhere in the sentence, are notoriously difficult to acquire, because learners attend to the most informative cue and neglect the others (DeKeyser, 2005; Ellis, 2006). Zero plurals combine both difficulties: the transparent mapping between form and meaning is missing on the noun, and the remaining cues are agreement markers that are redundant with one another. Overall, this literature provides a broader context for our finding that the efficient use of distributed agreement cues develops gradually and approaches native-like efficiency at approximately C1 level.

Together, the experiments point to stage- and domain-specific conflict resolution. Early processing is guided primarily by surface complexity and conceptual world knowledge—mechanisms that operate similarly in L1 and L2. Later integration depends more on overt morphological marking, with zero morphology posing a particular challenge for agreement integration and reanalysis in L2 and scaling with proficiency. The results provide initial evidence on how native speakers and L2 learners balance shared conceptual knowledge against language-specific structural cues.

Several extensions could test the generality and the underlying mechanisms of these findings. First, cross-linguistic comparisons that vary in gender systems and cultural stereotype strength are needed to determine to what degree the observed conceptual–lexical effects are language-independent or shaped by linguistic encoding. Investigating gender and number processing in typologically more dissimilar L1–L2 combinations and taking more fine-grained measures of participants’ multilingual experience into account would be particularly informative in this respect. Such work could also determine whether the reduced stereotype sensitivity reported for L2 processing (Jankowiak et al., 2025) is tied to specific L1–L2 constellations or reflects a more general property of non-native comprehension.

Second, extending the investigation to other types of zero-marking and underspecified morphology would clarify whether the observed reanalysis patterns reflect a broader sensitivity to missing overt cues rather than number-specific effects.

Finally, systematic manipulations of cue distribution and locality, combined with a more targeted investigation of subregional processing, would allow for direct tests of how readers prioritize and retrieve morphosyntactic cues under conditions of ambiguity. This applies in particular to our exploratory finding that L1 readers selectively target the determiner whereas L2 readers engage in a more distributed search, which should be examined more systematically to establish whether it reflects a general difference in cue-targeting mechanisms or a task-specific strategy. In addition, because the present data come from reading, the late effects reflect readers’ opportunity to regress to and re-inspect the noun phrase; in spoken comprehension, where the input is transient and externally paced, the same number ambiguity would have to be resolved from memory, which may increase processing costs, particularly for L2 listeners. Whether the present dissociation generalizes to auditory processing remains to be tested.

Taken together, the present results underscore that L1–L2 differences in sentence processing emerge primarily when overt morphological cues are absent rather than when comprehension relies on shared conceptual knowledge.

## Data Availability

The datasets presented in this study can be found in online repositories. The names of the repository/repositories and accession number(s) can be found at: OSF: https://doi.org/10.17605/OSF.IO/3K4WV.

## Footnotes

1 Digit span scores did not affect the results in any of the reported analyses.

2 Given this heterogeneity and our sample size, the resulting subgroups would have been too small to include these variables meaningfully in the statistical models, and a detailed table for each additional language would not allow reliable conclusions.

3 The feminine forms were derived with the suffix -in and the overt plurals with the suffixes -(*e*)n or -e (e.g., *Fotograf – Fotografin – Fotografen, Freund – Freundin – Freunde*). In all items except Kollege (*Kollegin, Kollegen*), both alternants are therefore one syllable longer than the masculine singular, whereas zero plurals are identical to the singular in spelling and syllable structure.

4 Feminine plural forms (e.g., *die Lehrerinnen* ‘the.PL teacher.F.PL’) were not included, as they mark gender (*−in*) and number (*−nen*) simultaneously; including them would have added a further condition to the design without contributing to either of the two contrasts of interest.

5
https://doi.org/10.17605/OSF.IO/3K4WV
