# Supplementary material for: Resolving conflicts during sentence processing in L1 and L2: eye-tracking evidence from gender stereotypes and zero plurals
Source: Front Psychol. 2026 Oct 27;17:1913837. doi: 10.3389/fpsyg.2026.1913837 (PMC13748372; doi:10.3389/fpsyg.2026.1913837)
Supplement: Supplementary file 1 [file Data_Sheet_1.PDF]

Supplementary Material

This supplement provides information on item selection and prestudies, as well as the detailed results of the significance tests for all generalized mixed models reported in the paper. Type II Wald χ^2 tests were used to assess the significance of main effects and interactions for all models (via the `Anova()` function of the `car` package in R).

The presentation order follows that of the results in the paper. To facilitate interpretation, each model output is accompanied by the corresponding plot (see figures in the paper).
For more information about the statistical procedures used, see the detailed R Markdown script that reproduces all the statistical analyses and plots. The script, along with the raw data, can be found in the public online repository.
Link: https://osf.io/3k4wv/overview?view_only=ac0f49f780ab4e568563e2769227fef7 .
(= anonymous link for peer review)

Contents

1 Information on item selection and prestudies 2
2 Gender manipulation 4
2.1 Gaze duration 4
2.2 Probability of regressions 5
2.3 Rereading times 6
3 Number manipulation 7
3.1 Gaze duration 7
3.2 Probability of regressions 8
3.3 Rereading times 9
3.4 Exploratory analyses – L1 & L2: sub-ROIs in plural contexts 10
3.5 Exploratory analyses – L2: LexTALE scores x sub-ROIs in plural contexts 11
3.6 Exploratory analyses – L2: LexTALE scores x plural morphology 12

1 Information on item selection and prestudies

All 32 experimental items were selected and controlled for factors including length, frequency, gender typicality, cognate status, and familiarity to the tested participant group (see Tables S1a and S1b). The materials were selected on the basis of several pretests. None of the participants who took part in the pretests participated in the final experiment. In the final experiment, the same 32 items were used in both the gender and number manipulations but were grouped differently according to the two levels of the respective manipulation: for gender, 16 gender-neutral and 16 male-biased items; for number, 16 zero-plural forms and 16 forms with overt plural marking. All other properties were kept constant across groups (see Tables S1a and S1b).

In a first pretest, the imageability and gender stereotypicality of role nouns were assessed. A total of 114 German nouns denoting persons were rated by 16 native speakers in an online questionnaire with randomized presentation order. For half of the participants, the masculine form was presented first, whereas the other half saw the feminine form first. To rate imageability, participants indicated on a seven-point Likert scale how easily the respective person (singular form, e.g., *Polizist / Polizistin*, '(male/female) police officer') could be visualized. To rate gender stereotypicality, following the questionnaire design of Misersky et al. (2014), participants estimated on an 11-point Likert scale the extent to which given groups of people (plural form, e.g., *Polizisten / Polizistinnen*, '(male/female) police officers') consisted of women and men (i.e., along a ratio ranging from 0 per cent male–100 per cent female to 100 per cent male–0 per cent female, or vice versa, with the midpoint representing a balanced gender ratio). Based on these ratings, 32 nouns were selected, of which 16 were rated high in imageability (mean = 5.18; SD = 0.64) and 16 low in imageability (mean = 2.52; SD = 0.78). Half of the nouns in each of these two subgroups were perceived as gender-neutral (mean = 52.08; SD = 5.97), whereas the other half were perceived as showing a clear male bias (mean = 34.26; SD = 4.61).

In a second pretest, familiarity among German learners at the target proficiency level of the study was assessed. Thirteen non-native German speakers at B2 level with various L1s provided information on whether they knew all critical nouns. They also provided feedback on how well they understood the sentences constructed for the study. On the basis of the learner feedback, minor adjustments were made to the materials to ensure that the final set of items contained only sentences that were readily understandable to L2 participants at B2 level.

Table S1a: Control criteria for the two groups of role nouns used in the gender manipulation (gender-neutral, male-biased).

	Gender unbalanced (male-biased) (N=16)			Gender balanced (N=16)			t-test		
**Variable**	**M**	**SD**	**Range**	**M**	**SD**	**Range**	**t**	**df**	**p**
Imageability rating	4.23	1.60	1.67–6.33	3.52	1.46	1.56–5.33	1.26	26.60	.218
Gender stereotype rating	34.24	4.61	27.5–41.3	52.08	5.97	43.3–63.3	-9.22	27.60	< .001***
Freq m	12.63	1.78	10–15	12.38	2.47	8–17	0.33	27.29	.746
Freq f	16.19	2.04	12–19	13.38	2.70	9–18	3.32	27.89	.003**
Length m	8.38	1.45	5–11	8.19	1.83	6–11	0.32	28.53	.751
Length f	10.38	1.45	7–13	10.13	1.89	8–13	0.42	28.14	.679

Note: Welch two-sample t-test. Significance levels: * $p < .05$, ** $p < .01$, *** $p < .001$.

Table S1b: Control criteria for the two groups of role nouns used in the number manipulation (zero-plurals, overt plural morpheme).

Plural Morpheme	Zero plural (N=16)			Overt plural (N=16)			t-test		
**Variable**	**M**	**SD**	**Range**	**M**	**SD**	**Range**	**t**	**df**	**p**
Imageability rating	4.02	1.55	1.67–6.22	3.70	1.56	1.56–6.33	0.56	27.53	.578
Gender stereotype rating	44.23	13.30	27.5–63.3	43.34	7.66	30–57.8	0.22	20.17	.829
Freq base noun	12.19	2.04	8–15	12.81	2.23	9–17	-0.83	29.77	.415
Length singular	8.38	1.50	6–11	8.19	1.80	5–11	0.32	29.07	.751
Length plural	8.38	1.50	6–11	9.88	2.00	6–13	-2.40	27.85	.023*

Note: Welch two-sample t-test. Significance levels: * $p < .05$, ** $p < .01$, *** $p < .001$.

2 Gender manipulation

2.1 Gaze duration

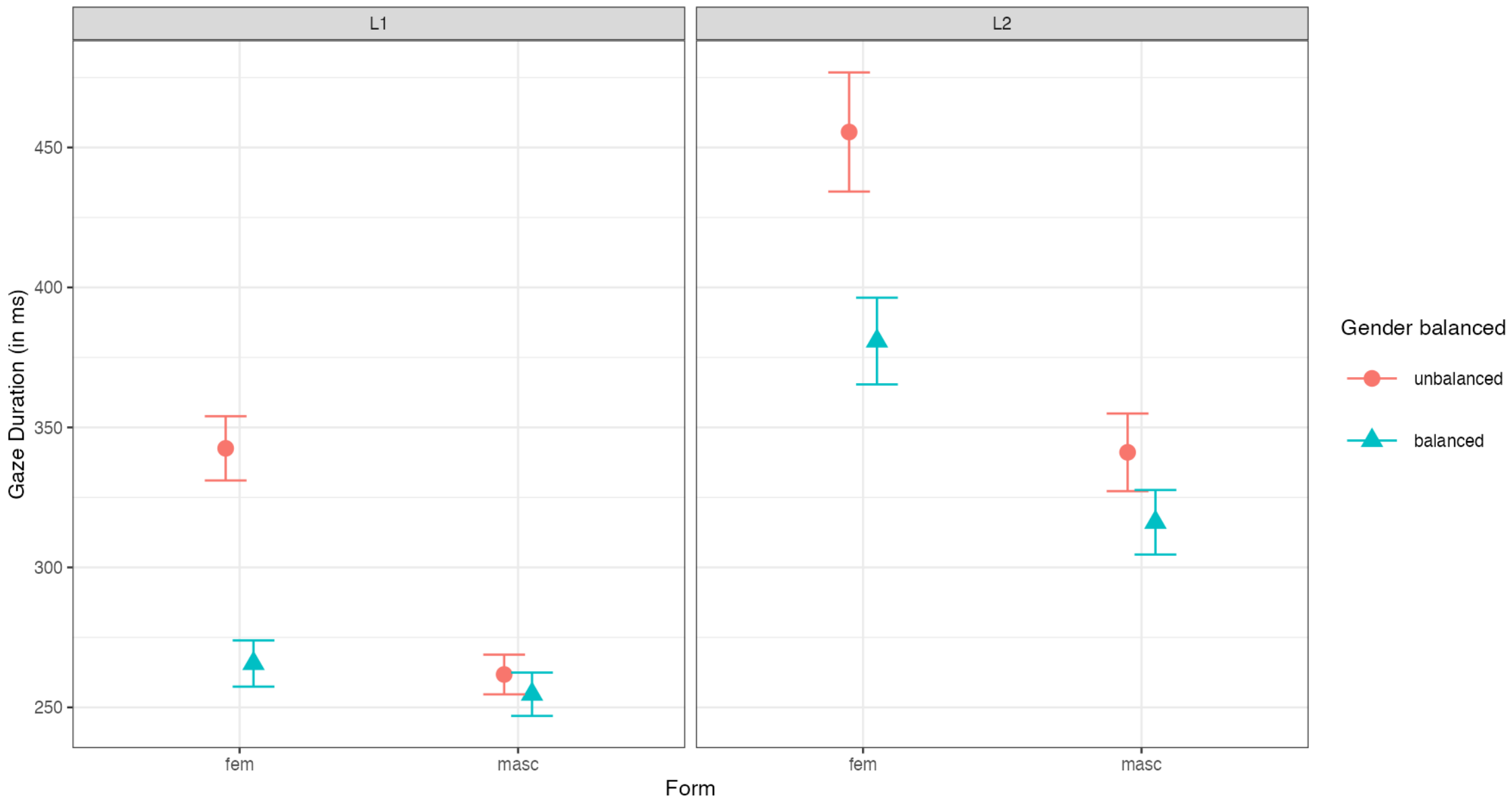

Supplementary Figure 1: Mean gaze durations in the gender experiment.

Table S2: Gender experiment – model output for gaze duration (ANOVA table, Type II)

Fixed Effects	Chisq	Df	Pr(>Chisq)	Signif
Language	31.896	1	<.001	***
Balancedness	6.297	1	.012	*
Form	104.785	1	<.001	***
Language:Balancedness	0.285	1	.594	
Language:Form	5.270	1	.022	*
Balancedness:Form	16.573	1	<.001	***
Language:Balancedness:Form	2.875	1	.090	

Model Structure:
GAZE_DURATION ~ Language * Balancedness * Form + (1 | participant_id) + (1 | item_id)
Significance levels: * p < .05, ** p < .01, *** p < .001

2.2 Probability of regressions

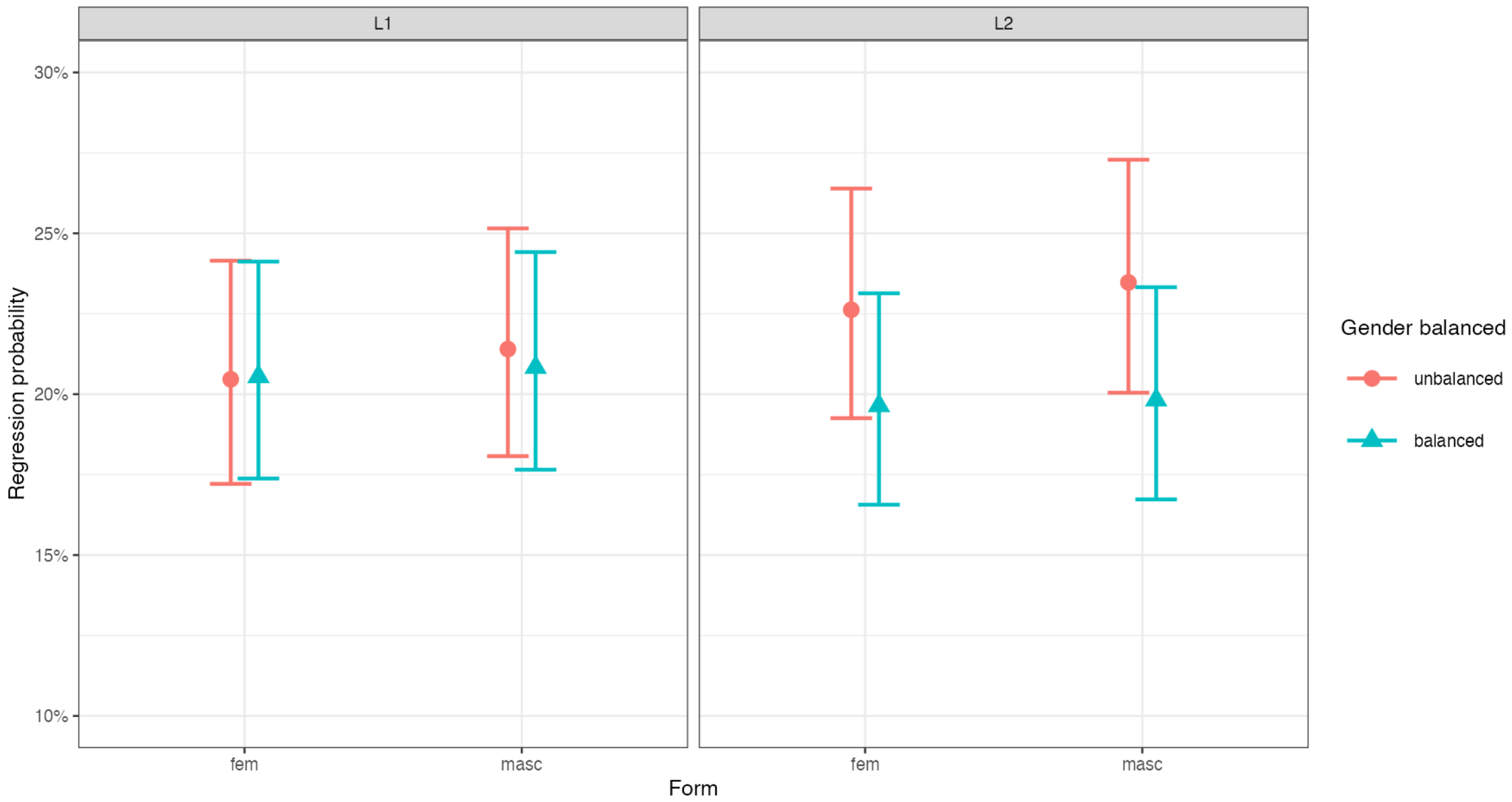

Supplementary Figure 2: Mean probabilities of regressions into critical ROIs in the gender experiment.

Table S3: Gender experiment – model output for regression probabilities (ANOVA table, Type II)

**Fixed Effects**	**Chisq**	**Df**	**Pr(>Chisq)**	**Signif**
Language	0.287	1	.592	
Balancedness	1.840	1	.175	
Form	0.113	1	.736	
Language:Balancedness	1.696	1	.193	
Language:Form	0.002	1	.968	
Balancedness:Form	0.021	1	.885	
Language:Balancedness:Form	0.000	1	1.000	

Model Structure:
Regression_Bin ~ Language * Balancedness * Form + (1 | participant_id) + (1 | item_id)
Significance levels: * p < .05, ** p < .01, *** p < .001

2.3 Rereading times

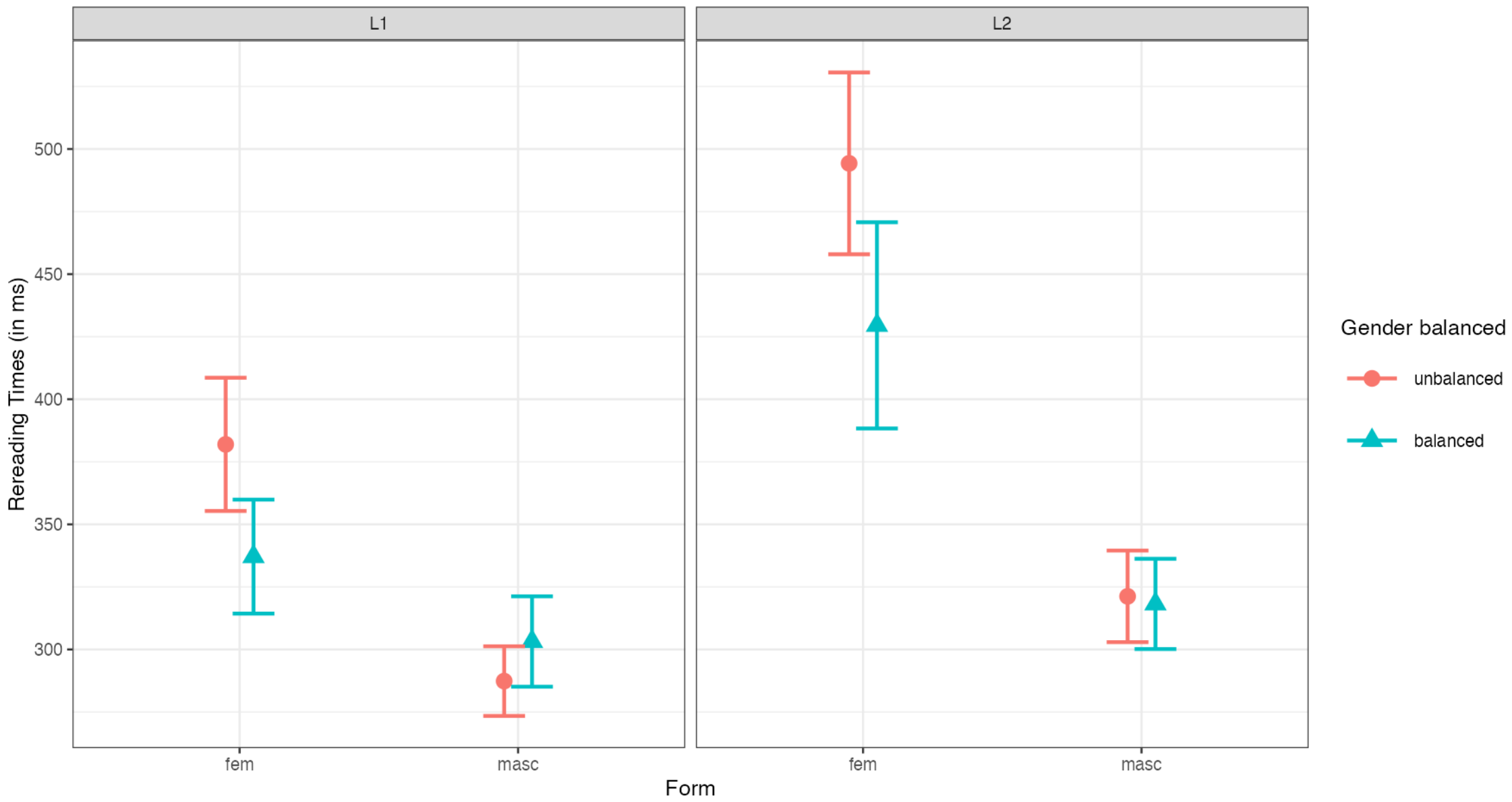

Supplementary Figure 3: Mean rereading times in the gender experiment.

Table S4: Gender experiment – model output for rereading times (ANOVA table, Type II)

Fixed Effects	Chisq	Df	Pr(>Chisq)	Signif
Language	6.108	1	.013	*
Balancedness	2.508	1	.113	
Form	54.751	1	<.001	***
Language:Balancedness	0.328	1	.567	
Language:Form	4.495	1	.034	*
Balancedness:Form	5.908	1	.015	*
Language:Balancedness:Form	0.057	1	.812	

Model Structure:
ReReading ~ Language * Balancedness * form + (1 | participant_id) + (1 | item_id)
Significance levels: * p < .05, ** p < .01, *** p < .001

3 Number manipulation

3.1 Gaze duration

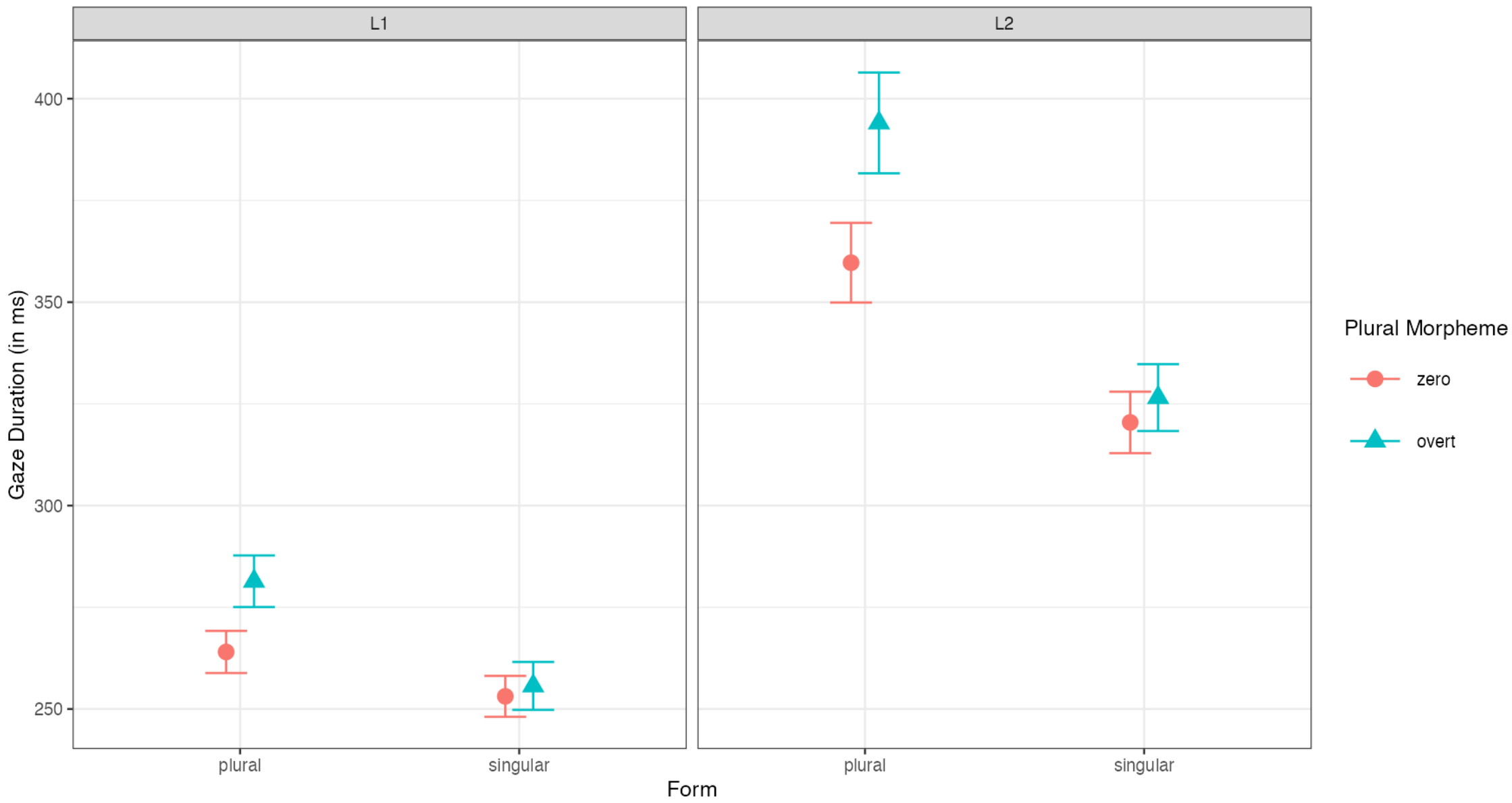

Supplementary Figure 4: Mean gaze durations in the number experiment.

Table S5: Number experiment – model output for gaze duration (ANOVA table, Type II)

**Fixed Effects**	**Chisq**	**Df**	**Pr(>Chisq)**	**Signif**
Language	57.548	1	<.001	***
Plural Morpheme	0.671	1	.413	
Form	59.323	1	<.001	***
Language:Plural Morpheme	0.008	1	.927	
Language:Form	6.443	1	.011	*
Plural Morpheme:Form	7.470	1	.006	**
Language:Plural Morpheme:Form	0.139	1	.710	

Model Structure:
GAZE_DURATION ~ Language * Plural Morpheme * Form + (1 | participant_id) + (1 | item_id)
Significance levels: * p < .05, ** p < .01, *** p < .001

3.2 Probability of regressions

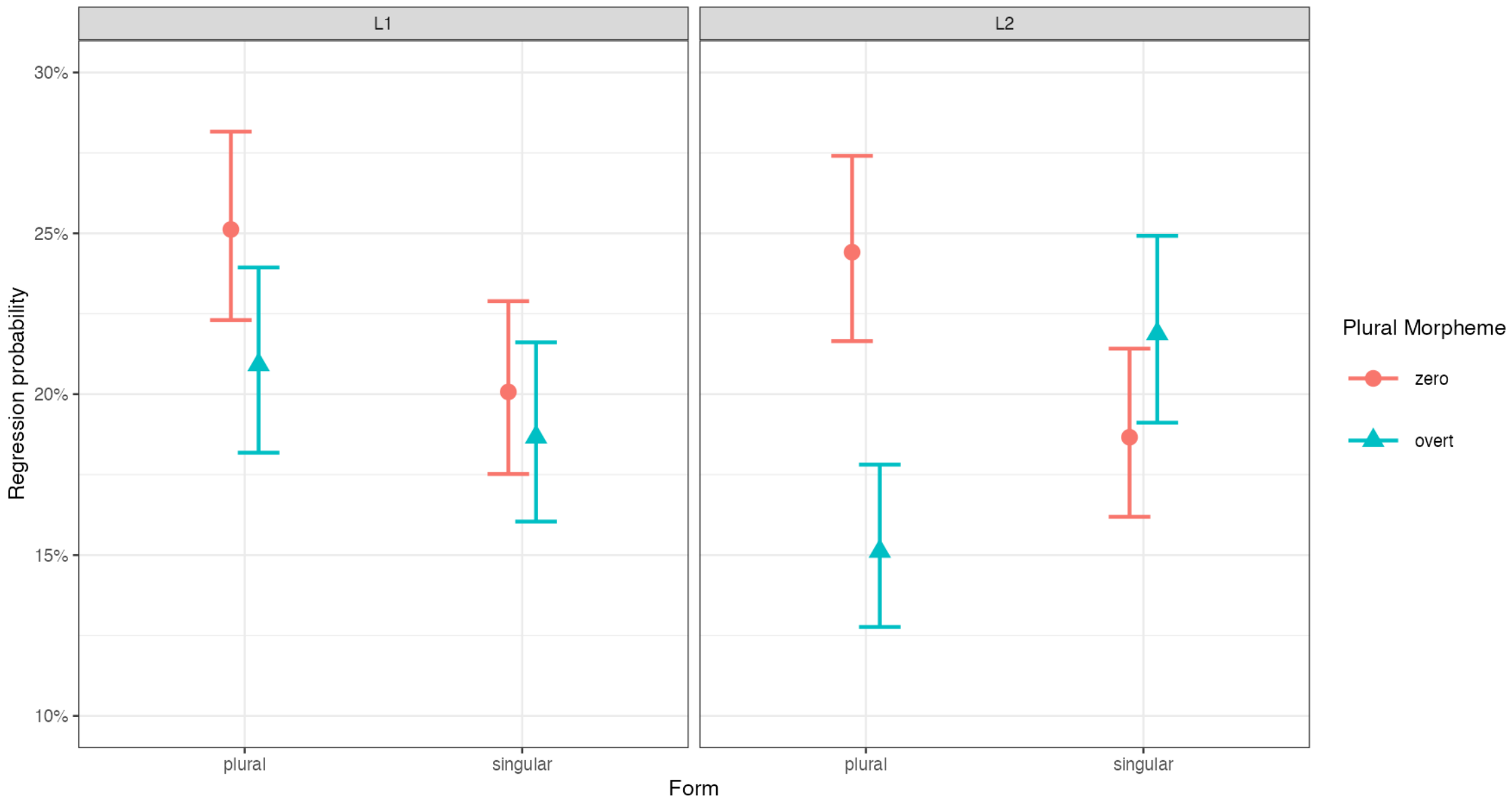

Supplementary Figure 5: Mean probabilities of regressions into critical ROIs in the number experiment.

Table S6: Number experiment – model output for regression probabilities (ANOVA table, Type II)

Fixed Effects	Chisq	Df	Pr(>Chisq)	Signif
Language	0.031	1	.861	
Plural Morpheme	4.920	1	.027	*
Form	2.993	1	.084	
Language:Plural Morpheme	0.360	1	.549	
Language:Form	4.119	1	.042	*
Plural Morpheme:Form	12.226	1	<.001	***
Language:Plural Morpheme:Form	2.527	1	.112	

Model Structure:
Regression_bin ~ Language * Plural Morpheme * Form + (1 | participant_id) + (1 | item_id)
Significance levels: * p < .05, ** p < .01, *** p < .001

3.3 Rereading times

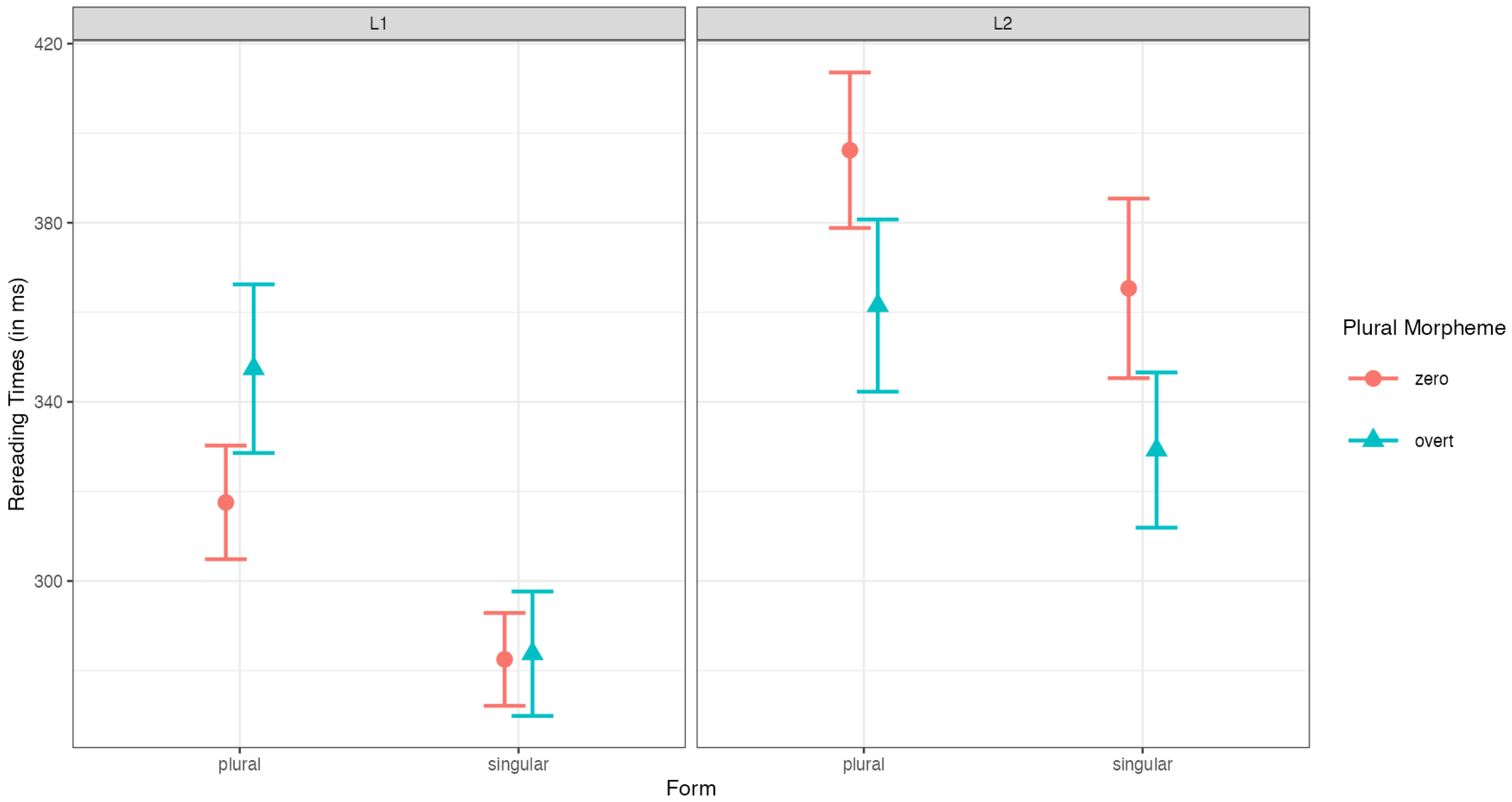

Supplementary Figure 6: Mean rereading times in the number experiment.

Table S7: Number experiment – model output for rereading times (ANOVA table, Type II)

Fixed Effects	Chisq	Df	Pr(>Chisq)	Signif
Language	10.348	1	.001	**
Plural Morpheme	0.730	1	.393	
Form	22.965	1	<.001	***
Language:Plural Morpheme	4.077	1	.043	*
Language:Form	1.219	1	.270	
Plural Morpheme:Form	2.717	1	.099	
Language:Plural Morpheme:Form	0.033	1	.856	

Model Structure:
ReReading ~ Language * Plural Morpheme * Form + (1 | participant_id) + (1 | item_id)
Significance levels: * p < .05, ** p < .01, *** p < .001

3.4 Exploratory analyses – L1 & L2: sub-ROIs in plural contexts

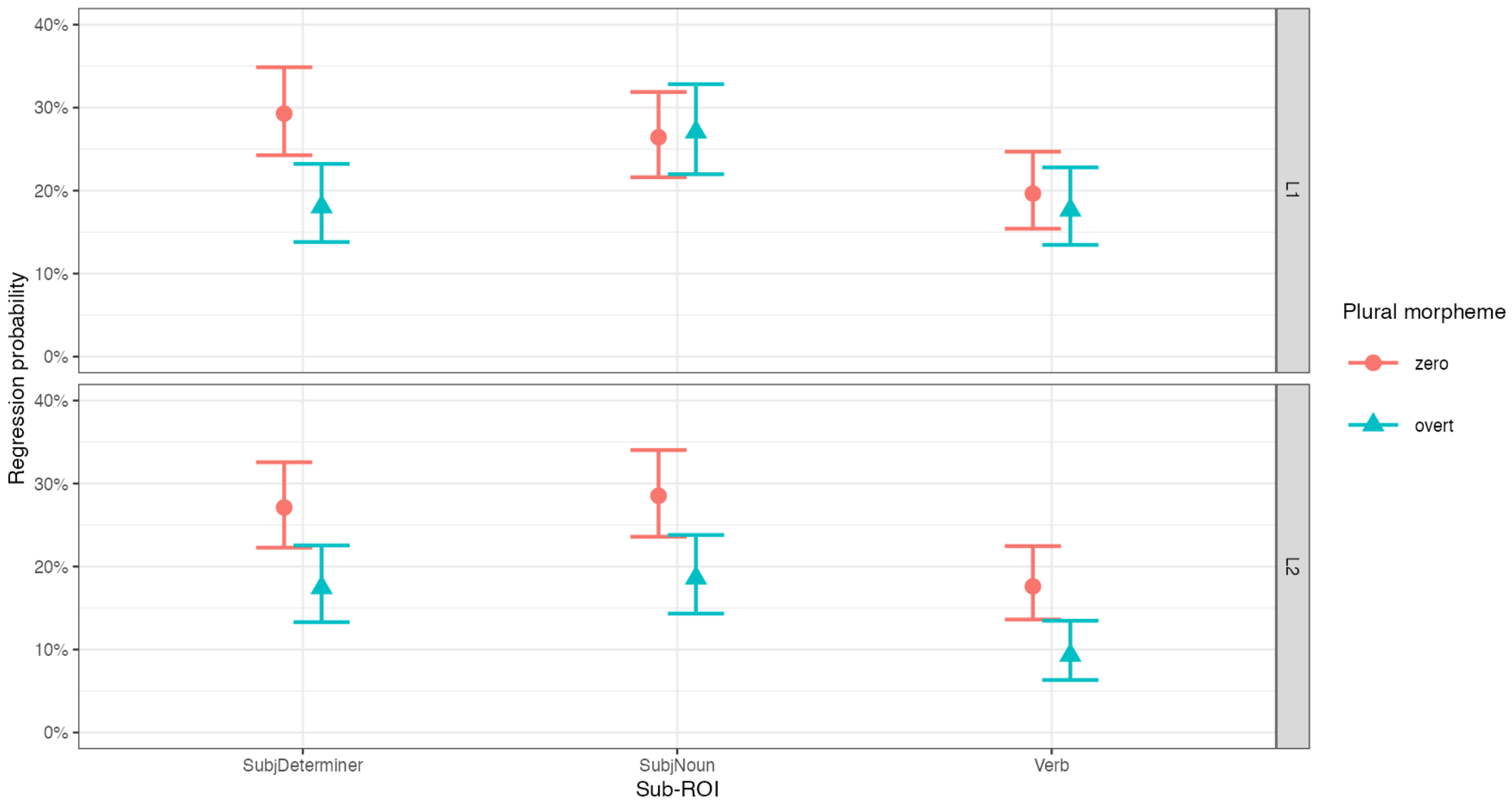

Supplementary Figure 7: Mean probability of regressions in plural contexts in the number experiment, split by sub-ROIs (target locations of regressions).

Table S8: Number experiment – **L1**: model output for regression probabilities in plural contexts by sub-ROIs (ANOVA table, Type II)

**Fixed Effects**	**Chisq**	**Df**	**Pr(>Chisq)**	**Signif**
Plural Morpheme	2.870	1	.090	
ROI	11.476	2	.003	**
Plural Morpheme:ROI	6.474	2	.039	*

Model Structure:
Regression_bin ~ Plural Morpheme * ROI + (1 | participant_id) + (1 | item_id)
Significance levels: * p < .05, ** p < .01, *** p < .001

Table S9: Number experiment – **L2**: model output for regression probabilities in plural contexts by sub-ROIs (ANOVA table, Type II)

**Fixed Effects**	**Chisq**	**Df**	**Pr(>Chisq)**	**Signif**
Plural Morpheme	13.425	1	<.001	***
ROI	22.479	2	<.001	***
Plural Morpheme:ROI	0.228	2	.892	

Model Structure:
Regression_bin ~ Plural Morpheme * ROI + (1 | participant_id) + (1 | item_id)
Significance levels: * p < .05, ** p < .01, *** p < .001

3.5 Exploratory analyses – L2: LexTALE scores x sub-ROIs in plural contexts

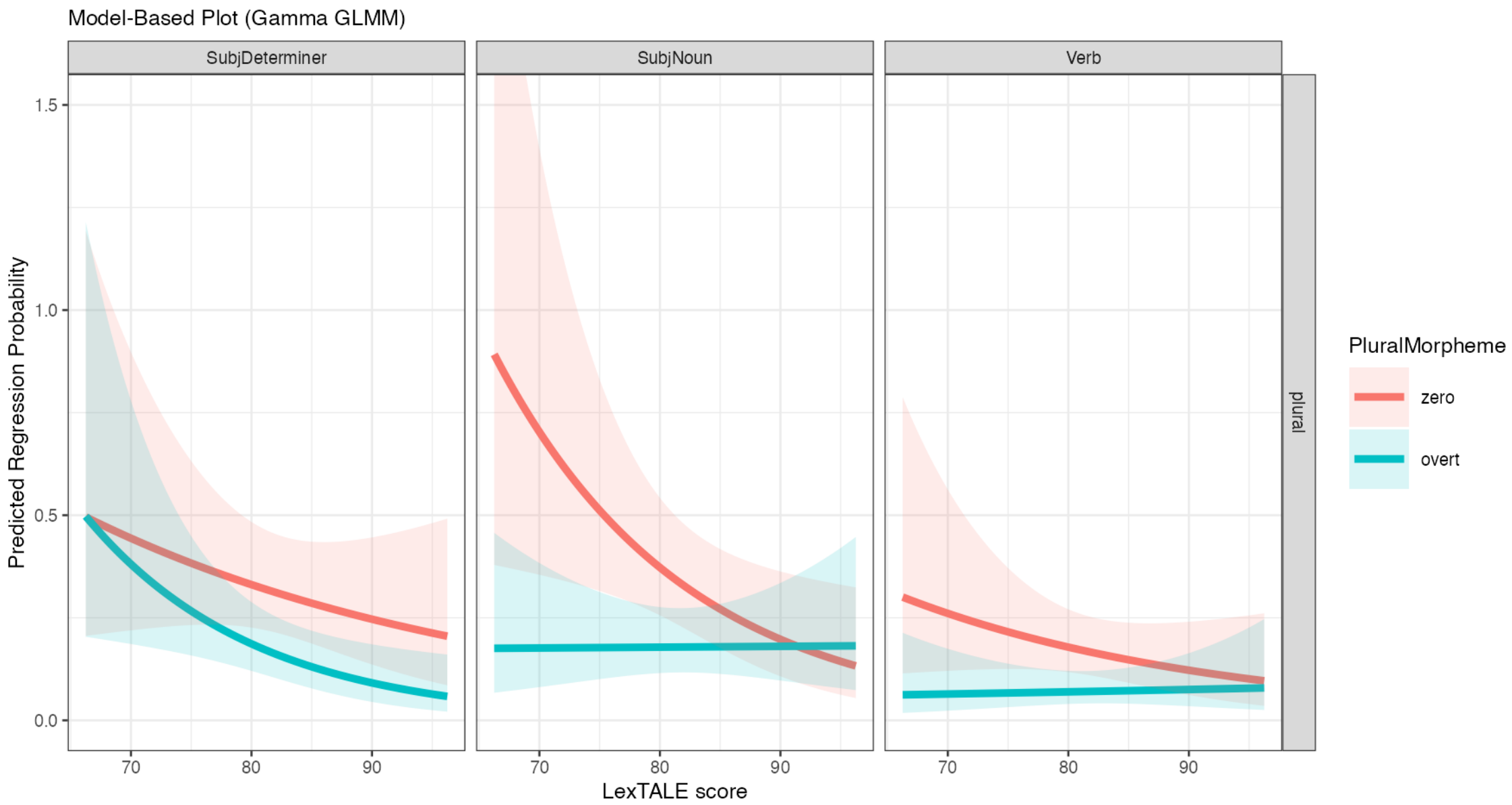

Supplementary Figure 8: Model-based predictions of probability of regressions for L2 participants in the number experiment. Influence of proficiency (LexTALE score) and plural morphology (overt vs. zero) at different sub-ROIs in plural contexts.

Table S10: Number experiment – model output for regression probabilities in plural contexts in L2 by sub-ROIs and LexTALE score (ANOVA table, Type II)

Fixed Effects	Chisq	Df	Pr(>Chisq)	Signif
LexTALE_NEW	4.251	1	.039	*
Plural Morpheme	13.449	1	<.001	***
ROI	22.809	2	<.001	***
LexTALE_NEW:Plural Morpheme	0.596	1	.440	
LexTALE_NEW:ROI	1.274	2	.529	
Plural Morpheme:ROI	0.702	2	.704	
LexTALE_NEW:Plural Morpheme:ROI	6.027	2	.049	*

Model Structure:
Regression_bin ~ LexTALE_NEW * Plural Morpheme * ROI + (1 | participant_id) + (1 | item_id)
Significance levels: * p < .05, ** p < .01, *** p < .001

3.6 Exploratory analyses – L2: LexTALE scores x plural morphology

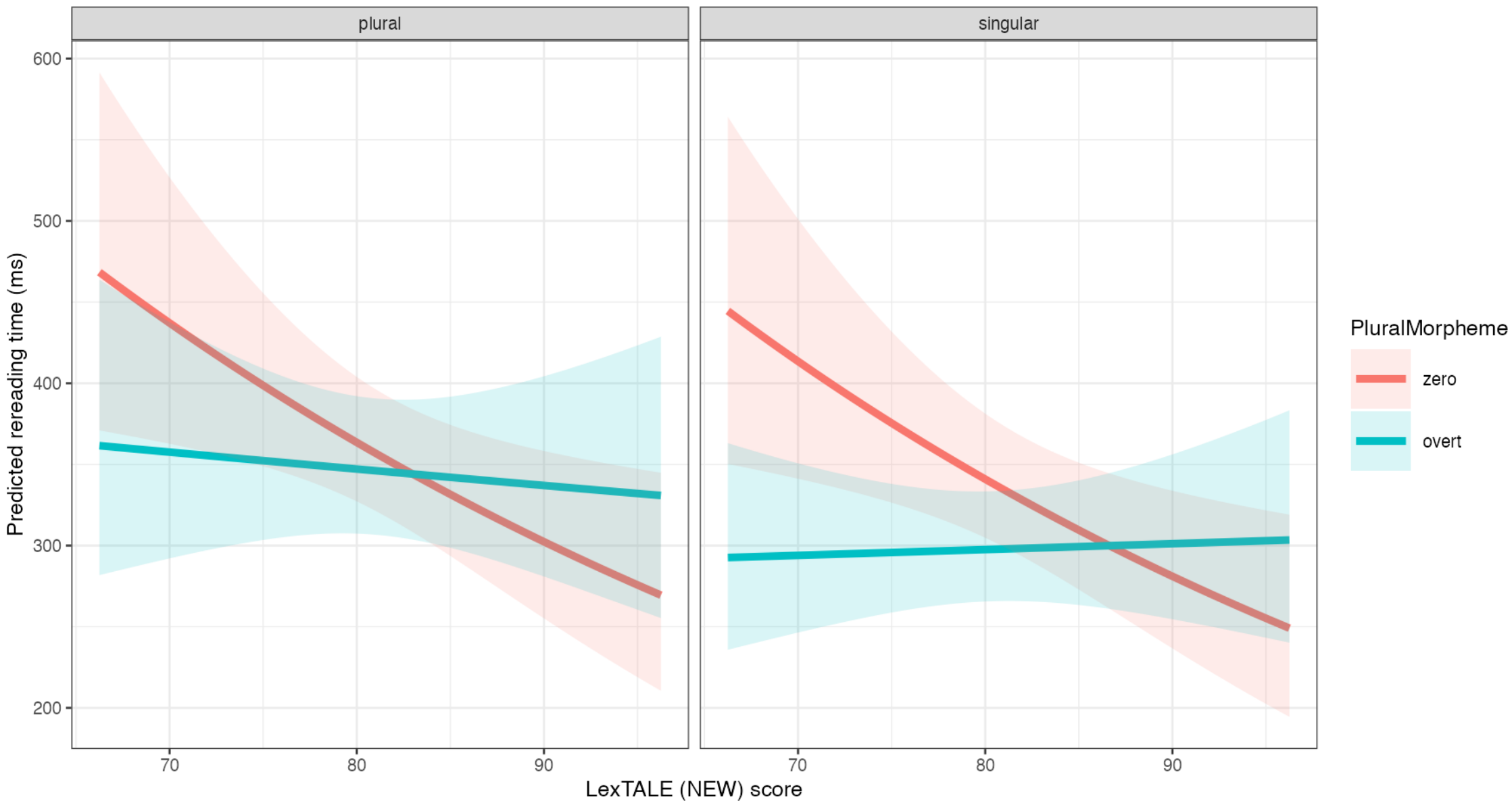

Supplementary Figure 9: Model-based predictions of rereading times for L2 participants in the number experiment. Influence of proficiency (LexTALE score) and plural morphology (overt vs. zero).

Table S11: Number experiment – model output for rereading times in L2 by LexTALE score (ANOVA table, Type II)

Fixed Effects	Chisq	Df	Pr(>Chisq)	Signif
LexTALE_NEW	3.736	1	.053	
Plural Morpheme	2.310	1	.129	
Form	6.776	1	.009	**
LexTALE_NEW:Plural Morpheme	7.646	1	.006	**
LexTALE_NEW:Form	0.105	1	.746	
Plural Morpheme:Form	1.076	1	.300	
LexTALE_NEW:Plural Morpheme:Form	0.169	1	.681	

Model Structure:
ReReading ~ LexTALE_NEW * Plural Morpheme * Form + (1 | participant_id) + (1 | item_id)
Significance levels: * p < .05, ** p < .01, *** p < .001
